# Erebosis, a new cell death mechanism during homeostatic turnover of gut enterocytes

**DOI:** 10.1371/journal.pbio.3001586

**Published:** 2022-04-25

**Authors:** Hanna M. Ciesielski, Hiroshi Nishida, Tomomi Takano, Aya Fukuhara, Tetsuhisa Otani, Yuko Ikegawa, Morihiro Okada, Takashi Nishimura, Mikio Furuse, Sa Kan Yoo

**Affiliations:** 1 Division of Developmental Biology and Regenerative Medicine, Kobe University, Kobe, Japan; 2 Physiological Genetics Laboratory, RIKEN CPR, Kobe, Japan; 3 Division of Cell Physiology, Kobe University, Kobe, Japan; 4 Laboratory for Homeodynamics, RIKEN BDR, Kobe, Japan; 5 Graduate School of Biostudies, Kyoto University, Kyoto, Japan; 6 Division of Cell structure, National Institute for Physiological Sciences, Okazaki, Japan; 7 Department of Physiological Sciences, School of Life Science, SOKENDAI, Okazaki, Japan; 8 Metabolic Regulation and Genetics, Institute for Molecular and Cellular Regulation, Gunma University, Maebashi, Japan; Institute of Cancer Research, Chester Beatty Laboratories, London, UNITED KINGDOM

## Abstract

Many adult tissues are composed of differentiated cells and stem cells, each working in a coordinated manner to maintain tissue homeostasis during physiological cell turnover. Old differentiated cells are believed to typically die by apoptosis. Here, we discovered a previously uncharacterized, new phenomenon, which we name erebosis based on the ancient Greek word erebos (“complete darkness”), in the gut enterocytes of adult *Drosophila*. Cells that undergo erebosis lose cytoskeleton, cell adhesion, organelles and fluorescent proteins, but accumulate Angiotensin-converting enzyme (Ance). Their nuclei become flat and occasionally difficult to detect. Erebotic cells do not have characteristic features of apoptosis, necrosis, or autophagic cell death. Inhibition of apoptosis prevents neither the gut cell turnover nor erebosis. We hypothesize that erebosis is a cell death mechanism for the enterocyte flux to mediate tissue homeostasis in the gut.

## Abstract 2

It has been believed that gut enterocytes continuously die through apoptosis. However, this study shows that gut enterocytes die through a novel cell death mechanism, named erebosis. Erobotic cells lack the characteristic features of apoptotic, necrotic or autophagic cell death; instead they lose their cytoskeleton, cell adhesion and organelles, and their nuclei become flat and indistinct.

## Introduction

Epithelial tissues are in a state of flux [1]. In the *Drosophila* gut, enterocytes, which absorb nutrition, are the major differentiated cell type [2]. The turnover rate under the physiological condition has been estimated from 4 days to 3 weeks [3–5]. Enterocytes have been described to die by apoptosis and to be apically extruded to the lumen during physiological homeostasis as well as under stress conditions such as infection and tissue damage [3,4,6–8]. In response to loss of enterocytes, intestinal stem cells (ISCs) proliferate to maintain the gut homeostasis [3]. On the other hand, it has been difficult to detect apoptosis reliably in the gut; thus, vital dyes such as SYTOX have been often used to detect dying cells [3,9], implying that alternative mechanisms might exist to regulate the gut cell turnover.

Here, we report the discovery of a previously unrecognized phenomenon in enterocytes, which are characterized by accumulation of Ance and loss of indispensable molecules and organelles. Ance is a *Drosophila* homolog of the mammalian Angiotensin-converting enzyme [10–13]. Although Angiotensin-converting enzyme is well known to convert angiotensin 1 to angiotensin 2 and to inactivate bradykinin, its substrate specificity is relatively low [14]. *Drosophila* Ance is secreted, and its substrates remain to be determined. Its expression has been described in imaginal cells (imaginal discs, abdominal histoblasts, gut imaginal cells, and imaginal salivary gland) [11], hemocyte progenitors [15], male accessory glands [16], and the testis [12]. Recently, Ance was shown to regulate imaginal disc development downstream of Dpp signaling [17].

## Results

**Fig 1:**
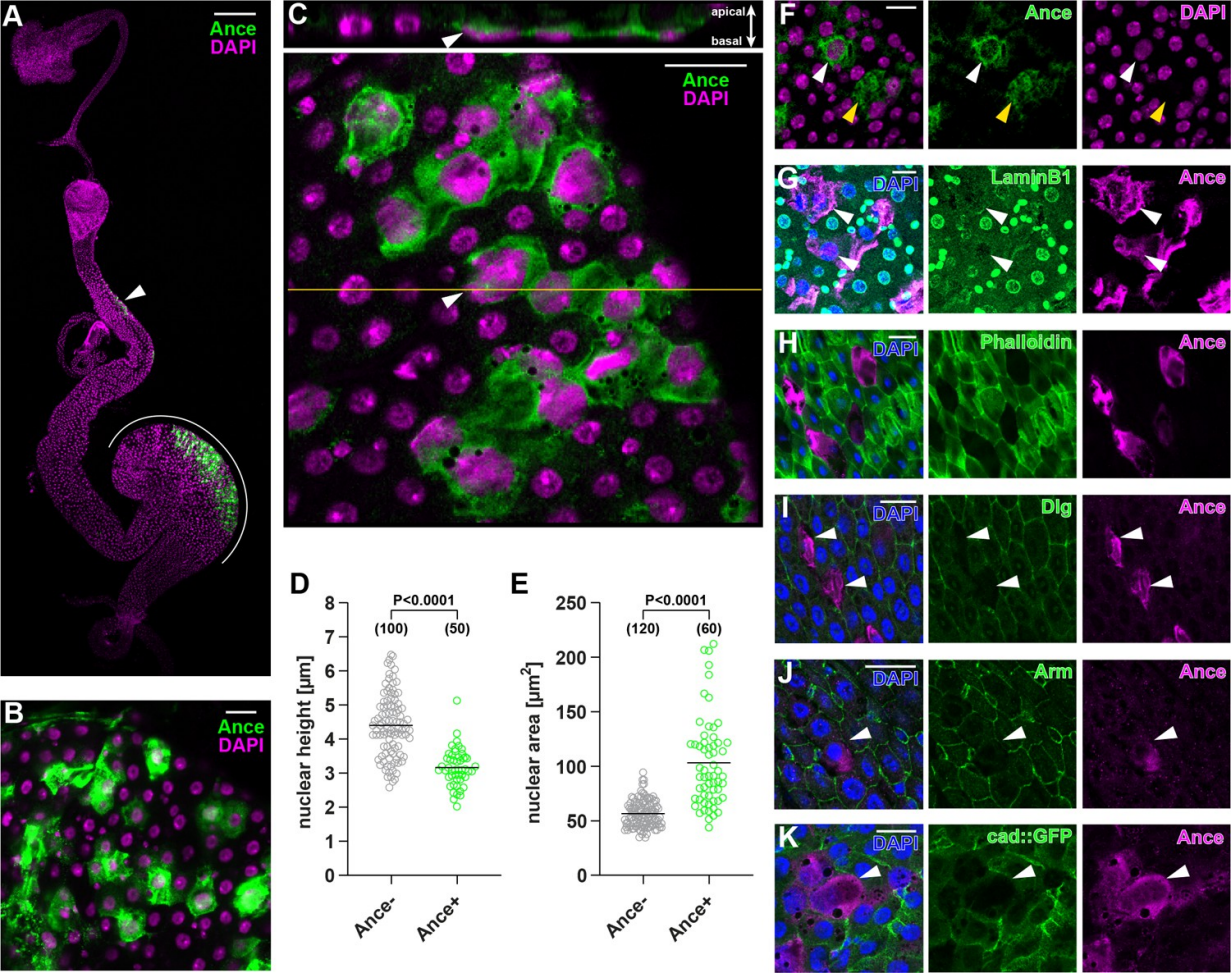
An Ance+ enterocyte subpopulation exhibits unique features. **(A)** Cells in R2 (arrowhead) and R4 (white line) express Ance. (**B)** An enterocyte subpopulation displays Ance protein. (**C)** Nuclei of Ance+ cells (arrowhead) are flattened and lie in close apposition to the basal, visceral muscle. (**D**-**E)** Quantification of nuclear height (D) and nuclear area (E). (**F)** Some Ance+ enterocytes show weak (white arrowhead) or absent (yellow arrowhead) genome staining by DAPI. (**G)** Immunostaining of nuclear LaminB1 is reduced in Ance+ enterocytes (arrowheads). (**H)** Ance+ enterocytes decrease F-actin indicated by the absence of phalloidin staining. (**I-K)** Ance+ enterocytes exhibit less cell adhesion components. Immunostaining for Dlg labeling septate junctions (I) and β-catenin/Arm labeling adherens junctions (J) was reduced in Ance+ cells (arrowheads). Likewise, cadherin GFP knock-in flies demonstrate less adherens junctions in Ance+ enterocytes (arrowhead) (K). Statistical significance was determined by using a two-tailed unpaired *t* test (D and E). S1 Data provides the source data used for all graphs and statistical analyses. Scale bars, 200 μm (A), 20 μm (B-C, F-K). Arm, Armadillo; Dlg, Discs large.

We serendipitously discovered that immunostaining of Ance demonstrates patchy labeling of gut enterocytes while looking at its expression patterns in diverse organs (Fig 1A and 1B). We detected Ance mainly in R1, R2, and R4 regions of the adult midgut (Fig 1A and 1B). Cell turnover is the most active in R4 (also known as P1 to P3) [2,3,18,19], and most research on the *Drosophila* midgut turnover focused on the R4 region or on the posterior midgut [3–5]. We also decided to focus on the R4 region in this study. Ance staining was specific because 2 *Ance* mutants [17] demonstrated no Ance immunostaining (S1A–S1D Fig). In addition to the patchy Ance staining within cells, we also noticed that there is a weak signal of Ance outside of the cell at the apical, luminal side, which reflects a secreted one (S1E Fig). During aging up to 3 weeks after eclosion, the number of Ance-positive cells remained constant (S1F Fig). We also confirmed that Ance-positive cells exist in the midgut of 2-month-old flies (S1G Fig). We observed similar expression patterns of Ance in both males and females (S1H Fig); thus, we used female flies for technical simplicity in this study.

We noticed that cells that have Ance possess several unusual characteristics. They are usually flat and lie in close apposition to the basal, visceral muscle layer (Fig 1C and 1D). The nuclear diameter tends to be larger than surrounding enterocyte neighbors (Fig 1E). Occasionally, genome staining with DAPI or Hoechst was weak, or disappeared, in an extreme case (Figs 1F and S1I), suggesting that they either lose a DNA content or adopt a closed chromatin structure that makes the dyes inaccessible. The nuclear lamin is also reduced in Ance-positive cells (Figs 1G and S1J). Cells with Ance have a low amount of actin filament (Figs 1H and S1K). They also have less amounts of septate junctions and adherens junctions (Figs 1I, 1J, S1L and S1M). This was not due to an artifact of immunostaining, since cad-GFP also demonstrates a reduced amount of adherens junctions in cells with Ance (Figs 1K and S1N).

*Myo1D-Gal4* has been extensively used as a pan enterocyte driver in the field. We examined whether *Myo1D-Gal4* promotes expression of GFP in Ance-positive cells.

**Fig 2:**
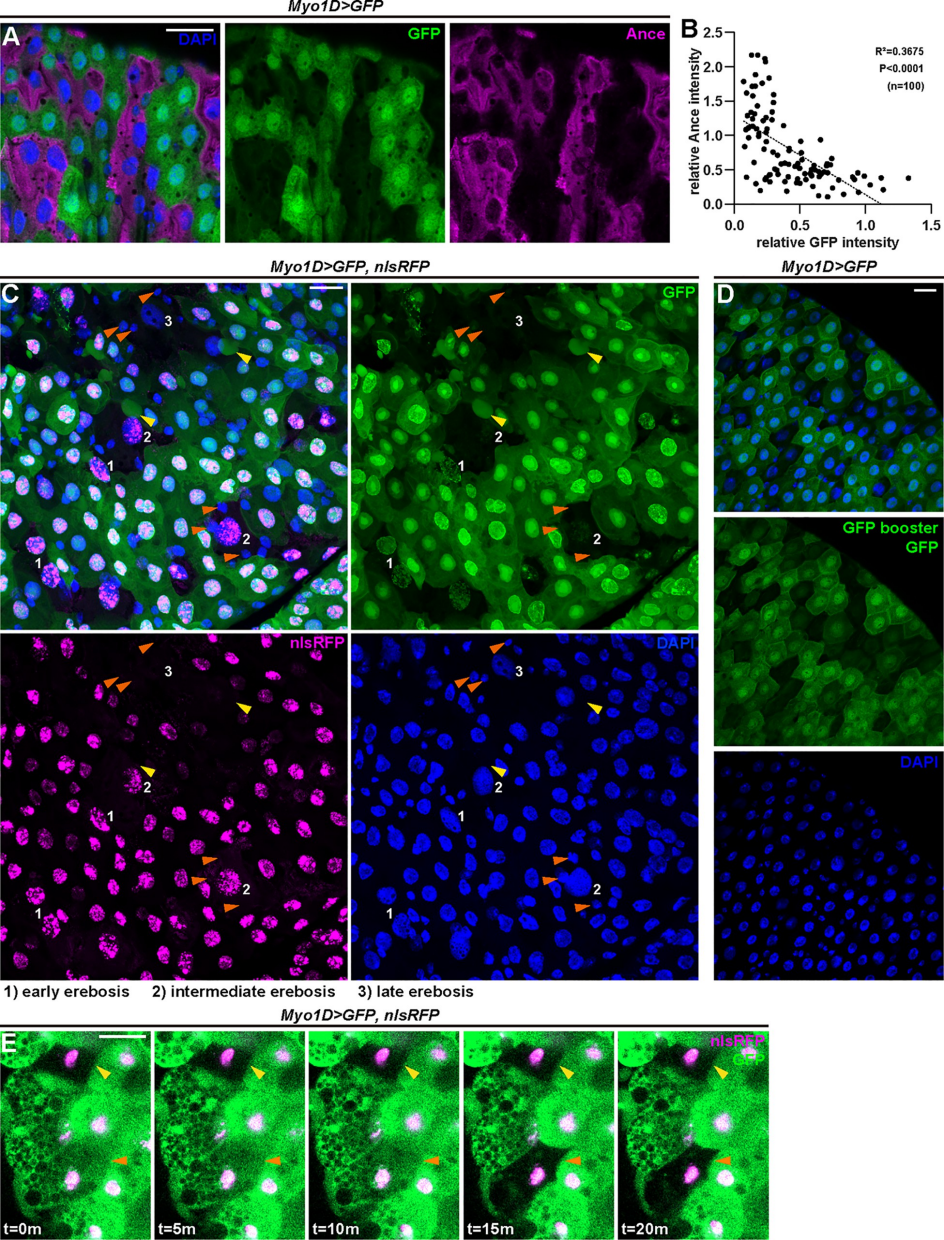
Ance+ enterocytes undergo erebosis. **(A)** Ance+ immunostaining displays an inverse relationship to *Myo1D*-driven GFP fluorescence. Erebotic cells are labeled by Ance or loss of GFP signals. (**B)** A correlation analysis of median fluorescence intensity of Ance and GFP in the cell cytoplasm indicates that enterocytes exhibiting high Ance signals show low GFP signals and vice versa. Pearson’ correlation coefficient (R) was calculated: R = −0.6062, R^2^ = 0.3675, *P* < 0.0001. (**C)** *Myo1D*-driven GFP and nuclear RFP reveal a gradual process of erebosis. At early erebosis (1), cells lose cytoplasmic GFP but retain nuclear GFP and RFP. At intermediate erebosis (2), cells do not have signals of GFP but still retain nuclear RFP. At late erebosis (3), cells lose both GFP and RFP signals. Erebosis-induced protrusion can be observed in enterocytes near erebotic cells (yellow arrowheads). Small cells adjacent to erebotic cells are frequently visible (orange arrowheads). (**D)** GFP protein was not detectable by a GFP nanobody in erebotic enterocytes lacking *Myo1D*-driven GFP fluorescence. (**E)** Time-lapse imaging demonstrates that loss of GFP occurs in a live condition. The yellow arrowhead indicates a cell that had already undergone erebosis at the time of imaging. The orange arrowhead indicates a cell that lost cytoplasmic GFP during imaging. The loss of GFP occurred within 5 minutes. S1 Data provides the source data used for all graphs and statistical analyses. Scale bars, 20 μm.

Intriguingly, we did not detect GFP signals in all enterocytes with *Myo1D-Gal4* (Fig 2A). We found that Ance and *Myo1D-Gal4*–driven GFP demonstrate a complementary pattern, that is, an inverse relationship (Fig 2A and 2B). This suggests that either *Myo1D-Gal4* does not drive GFP expression in Ance-positive cells or that GFP is denatured, degraded, or somehow lost. Ance-positive cells could not be labeled with GFP driven by *actin-Gal4*, *tub-Gal4*, or another enterocyte driver *Npc1b-Gal4* (S2A–S2C Fig).

At this moment, due to the peculiarity of Ance-positive cells (weak nuclear staining, flat and large nucleus, loss of F-actin and junction components, loss of GFP signals), we named this phenomenon erebosis, based on the ancient Greek word έρεβος [erebos] meaning deep darkness.

Intriguingly, when we expressed both GFP and nlsRFP using *Myo1D-Gal4*, we noticed that erebotic cells (cytoplasmic GFP-negative cells) usually have nlsRFP signals (Fig 2C). This indicates that *Myo1D* does induce expression of *Gal4* in erebotic cells but that erebotic cells lose GFP signals. Close examination showed that there are 3 types of erebotic cells (cytoplasmic GFP negative) with different amounts of nuclear GFP and nuclear RFP. The first one retains nuclear RFP and faint signals of nuclear GFP. The second one has only nuclear RFP without nuclear GFP. The third one has lost both nuclear RFP and nuclear GFP. Assuming that losing fluorescence is a gradual process, we reason that, during erebosis, cells first lose cytoplasmic GFP, then nuclear GFP, followed by loss of nuclear RFP. Longer retention of nuclear RFP signals compared to nuclear GFP is likely due to the stability difference of the fluorescent proteins. For example, RFP is more resistant to low pH than GFP [20]. Similar to the case of nuclear GFP and RFP, cytoplasmic RFP became reduced but persisted longer than cytoplasmic GFP in erebotic cells (S2D and S2E Fig). As a typical feature of erebosis, we also noticed that enterocytes surrounding the erebotic cells often protrude toward erebotic cells (Fig 2C).

Since GFP signals are lost in erebotic cells, we investigated whether loss of GFP signals is due to protein denaturing, for example, in an acidic environment within erebotic cells, or due to bona fide loss of protein. If denaturing leads to loss of GFP signals, a GFP antibody should still be able to detect GFP in erebotic cells. We could not detect GFP in erebotic cells even with a GFP nanobody (Fig 2D), suggesting that GFP is likely lost rather than being denatured in erebotic cells.

To clarify that loss of GFP is not an artifact of fixation, we visualized erebotic cells and the moment of erebosis performing live imaging. We readily observed erebotic cells in a live condition (Fig 2E and S1 Movie). Time-lapse live imaging revealed that loss of cytoplasmic GFP occurs fast (within 5 minutes) (Fig 2E and S1 Movie). The nuclear signals of GFP and RFP persisted relatively long time, at least 12 hours after the loss of cytoplasmic GFP. Live imaging definitively demonstrates that loss of GFP is not an artifact of fixation, as occurring in a live setting.

**Fig 3:**
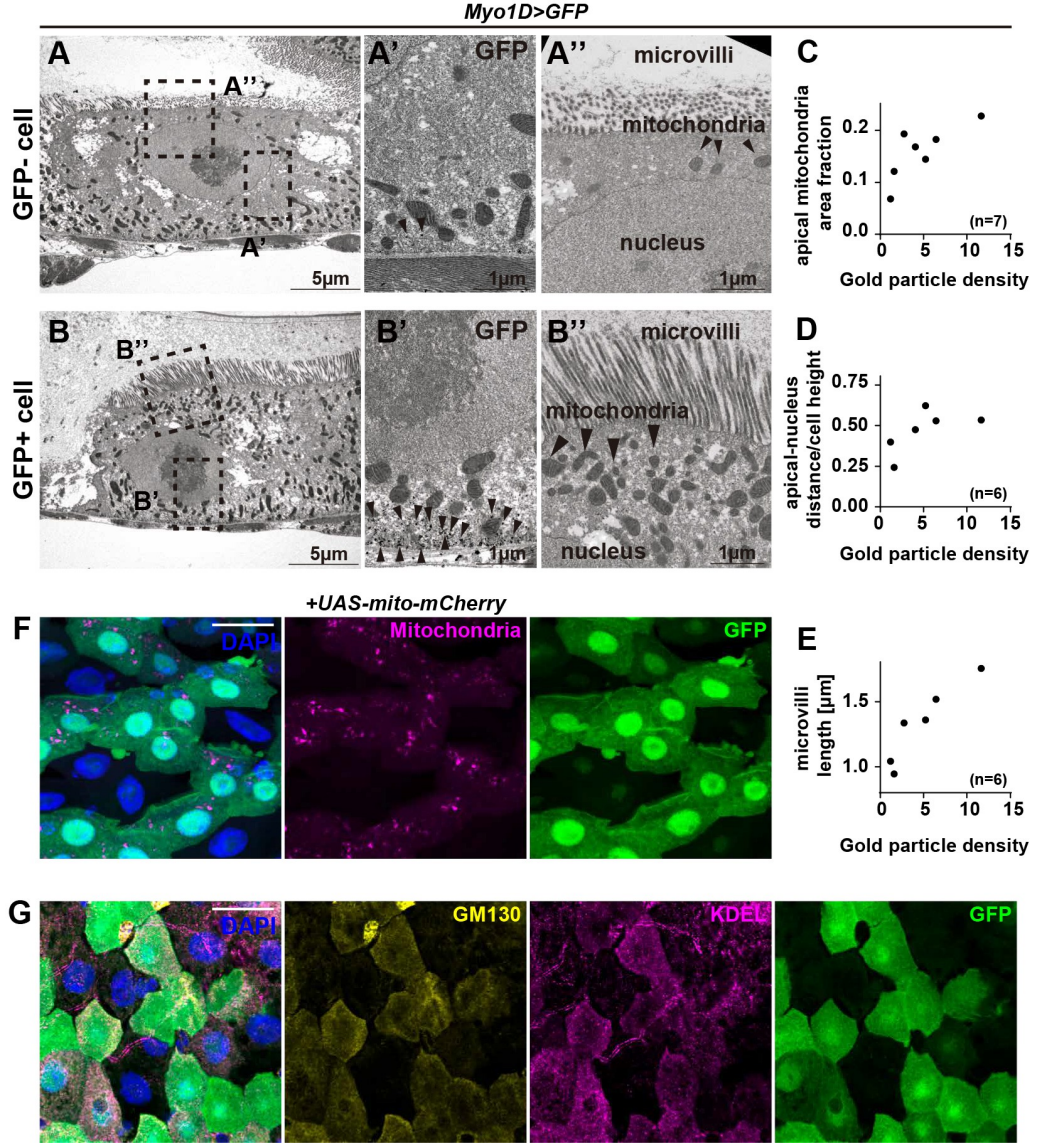
Erebotic cells lose organelles. **(A)** Low magnification transmission electron micrograph of GFP-negative (GFP−) cells. Squares indicate the regions shown in high-magnification images. (**A’)** The enlarged image of the basal region of GFP− cells shows sparse labeling by anti-GFP (arrowheads). (**A”)**. The enlarged image of the apical region of GFP− cells shows short microvilli, a reduced number of mitochondria, and reduced apical cytoplasm. (**B)** Low magnification transmission electron micrograph of GFP-positive (GFP+) cells. Squares indicate the regions shown in high-magnification images. (**B’)** The enlarged image of the basal region of GFP+ cells shows numerous labeling by anti-GFP (arrowheads). (**B”)** The enlarged image of the apical region of GFP+ cells shows well-developed microvilli and the apical cytoplasm rich with mitochondria. (**C)** Quantitation of apical mitochondria area fraction shows fewer apical mitochondria in GFP− cells. (**D)** Quantitation of the ratio of apical membrane–nucleus distance and cell height shows reduced apical cytoplasmic content in GFP− cells. (**E)** Quantitation of microvilli length shows shorter microvilli in GFP− cells. (**F)** Erebotic cells exhibit a reduced number of mitochondria. (**G)** Immunostaining for the *cis*-Golgi marker GM130 and for the ER marker KDEL shows that erebotic cells have very few of these organelles. S1 Data provides the source data used for all graphs. Scale bars, 5 μm (A, B), 1 μm (A’, A”, B’, B”), 20 μm (F, G).

Taking advantage of GFP loss in erebotic cells, we observed the ultrastructure of erebotic cells by performing immuno-electron microscopy (immuno-EM) with a GFP antibody in the gut where *Myo1D-Gal4* drives GFP (Fig 3A–3E). Cells with a low amount of GFP show a reduced number of mitochondria and a reduced cytoplasmic content above the nucleus (Fig 3C and 3D). We also observed that these cells have shorter microvilli (Fig 3E). The immuno-EM data prompted us to examine organelles in erebotic cells with fluorescent microscopy. Consistent with the immuno-EM data, we found that erebotic cells have a reduced number of mitochondria (Fig 3F). They also have reduced amounts of ER and Golgi (Fig 3G).

**Fig 4:**
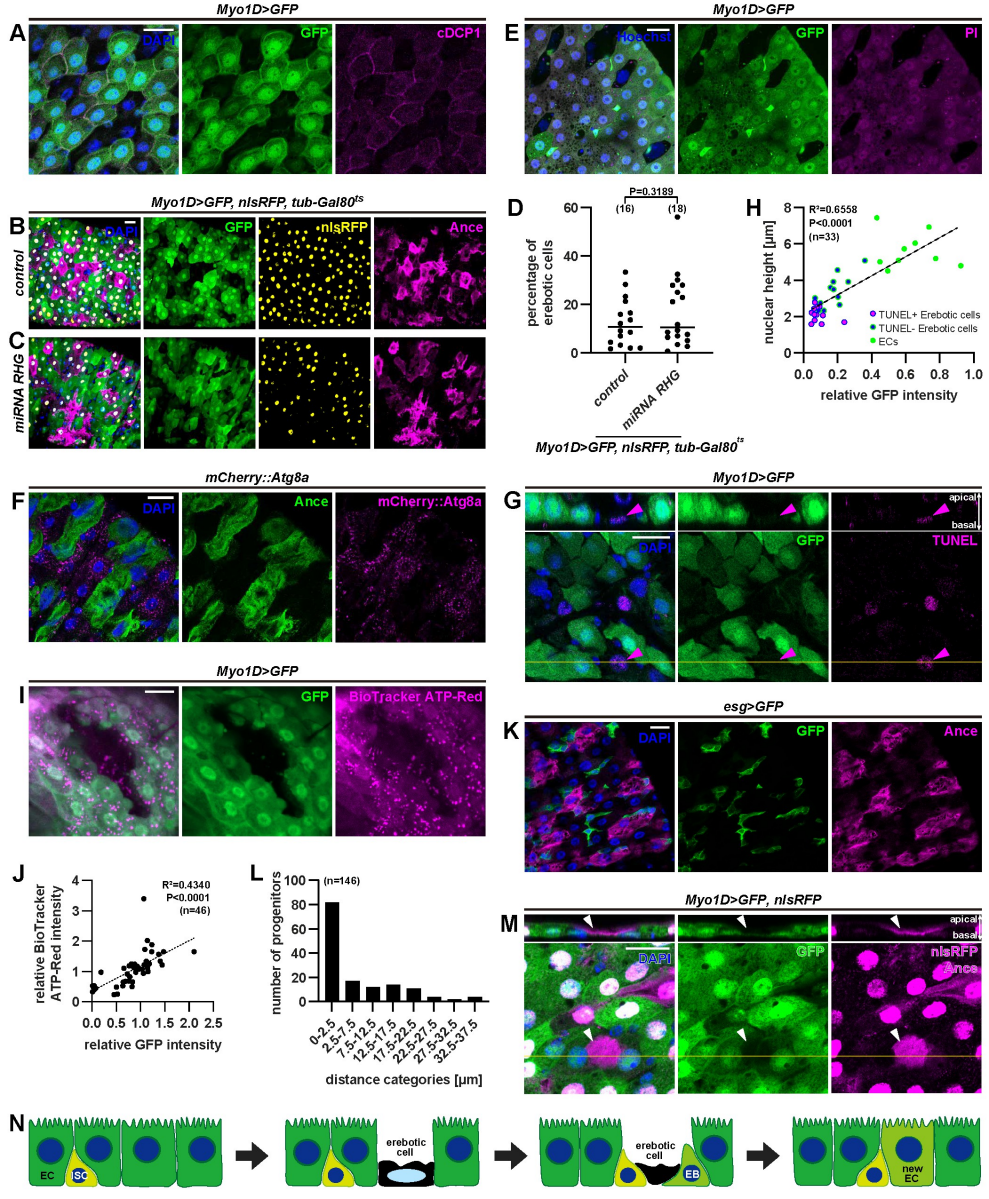
Erebosis is unprecedented cell death. **(A)** Immunostaining for cleaved caspase 1 (cDCP1) does not show any increased signal in erebotic cells marked by absence of GFP fluorescence. (**B, C)** Erebotic cells indicated by absence of *Myo1D*-driven GFP and presence of Ance staining are visible even with suppression of apoptosis by expression of miRNA against *rpr*, *hid*, and *grim*. (**D)** Quantification of the percentage of erebotic cells with/without miRNA for *rpr*, *hid*, and *grim*. (**E)** Live imaging after PI feeding demonstrates that PI cannot enter erebotic cells. (**F)** Autophagosomes labeled by mCherry::Atg8a are reduced in erebotic (Ance+) cells. (**G)** Erebotic cells with a lower nuclear height and less GFP signals are labeled by TUNEL staining (arrowhead). (**H)** Correlation analysis of the nuclear height and nuclear GFP intensity demonstrates that cells at late erebosis (shorter nuclear height and weaker nuclear GFP signals) tend to be TUNEL positive. Pearson’ correlation coefficient (R) was calculated: R = 0.8098, R^2^ = 0.6558, *P* < 0.0001. (**I)** Erebotic cells have reduced amounts of cellular ATP labeled by BioTracker ATP-red, indicating their reduced metabolic activity. Note that the punctate signals of BioTracker ATP-red likely represent ATP in mitochondria. (**J)** Correlation analysis of ATP detected by BioTracker ATP-red and GFP intensity. Pearson’ correlation coefficient (R) was calculated: R = 0.6588, R^2^ = 0.4340, *P* < 0.0001. (**K)** Progenitor cells labeled by *escargot*-driven GFP (*esg*>GFP) are present in close proximity to Ance+ enterocytes. (**L)** Distance between erebotic cells and progenitor cells. (**M)** Immunostaining for Ance shows that a cytoplasmic legacy of an erebotic cell (arrowhead) is being pushed up by 2 enterocytes. The 2 enterocytes are considered to be relatively young because they have only GFP expression, not nlsRFP, and maturation of GFP is faster than of RFP. This captures a potential moment of the cell replacement. (**N)** Schematic of the erebosis process. Erebotic cells lose cytoskeleton, adhesion components, and organelles. Erebotic cells eventually become TUNEL positive, die, and are replaced by new enterocytes. Statistical significance was determined by using a two-tailed unpaired *t* test (D). S1 Data provides the source data used for all graphs and statistical analyses. Scale bars, 20 μm. EB, enteroblast; EC, enterocyte; ISC, intestinal stem cell; PI, propidium iodide.

What is the function of erebosis? The loss of fundamental cellular components such as organelles, cytoskeleton, junctions, the nuclear membrane, and, possibly, DNA made us to imagine that this is some type of cell death. At least, it is difficult to imagine that erebotic cells are maintaining life with active metabolism. In general, there are 3 pathologically categorized types of cell death: apoptosis, necrosis, and autophagic cell death. We could not detect caspase activation in erebotic cells based on cleaved DCP1(Fig 4A). Caspase inhibition by p35, miRNAs for *rpr*, *hid*, and *grim* or *dpf* mutation did not suppress erebosis either (Figs 4B–4D and S3A–S3E). Clonal analyses of homozygous *H99* or the *Dronc* null mutation, both of which suppress apoptosis [21–23], demonstrated that erebosis occurs in the mutant clones (S3F–S3H Fig). Erebotic cells did not have any feature that occurs with infection-induced cell shedding, such as up-regulation of *upd2*, or involvement of JNK or IMD pathways [6] (S4A–S4F Fig). Erebosis was also observed in a sterile condition, where flies were bleached when they were embryos and cultured with antibiotics (S4G–S4J Fig). A necrosis marker, propidium iodide, which can enter cells when the plasma membrane is breached, did not enter erebotic cells (Fig 4E). An autophagy marker (mCherry-Atg8a) did not label erebotic cells either (Fig 4F). Autophagy inhibition through knockdown of *Atg* genes did not suppress erebosis (S5 Fig).

We investigated whether a general cell death marker TUNEL, which detects the DNA nick, labels erebotic cells. TUNEL is known to label not only apoptotic cells but also other cell death such as necrotic cells [24–26]. TUNEL did not label all erebotic cells, but labeled ones with the shorter nucleus height and lower GFP signals, which we found correlate (Fig 4G and 4H), suggesting that TUNEL labels cells at late erebosis. Inhibition of caspase-activated DNAse affected neither TUNEL nor erebosis (S6A–S6E Fig), consistent with the idea that erebosis is a process that is distinct from apoptosis. These observations suggest that erebosis is a gradual process toward cell death, which eventually leads to nicking and degradation of DNA. To further investigate the idea that erebosis is a mechanism toward cell death, we examined indispensable “house-keeping” molecules in addition to the features we already characterized in erebotic cells. We found that erebotic cells lose tubulin in addition to F-actin, and Fibrillarin, an important nucleolus component (S6F and S6G Fig). They also have a reduced amount of cellular ATP (Fig 4I and 4J). In sum, erebosis does not have any traditional feature of apoptosis, necrosis, or autophagy, and we speculate that it is a process toward cell death.

Since erebotic cells were only positively labeled with Ance, we investigated the role for Ance in erebosis. We confirmed that 2 RNAis driven by *Myo1D-Gal4* decrease signals of Ance (S7A–S7D Fig). Interestingly, knockdown of *Ance* with these RNAis did not affect erebosis (S7A–S7C and S7E Fig). *Ance* mutation did not affect the gut cell turnover detected by the mitotic activity of ISCs (S7F Fig). *Ance* overexpression did not affect erebosis (S7G–S7J Fig). We found that Ance inhibition in enterocytes affects the amount of excreted waste that is labeled intensely with bromophenol blue (S7K–S7M Fig). Since Ance does not affect erebosis itself, we interpret that the effect of Ance inhibition on excretion is not due to erebosis, but due to another mechanism that Ance itself mediates. One interesting note on Ance is that its transcription detected by *Ance-Gal4*–mediated GFP was also observed in the cells surrounding erebotic cells (S7N and S7O Fig). Thus, non-erebotic, normal enterocytes also express some amount of *Ance*. This constitutive expression of *Ance* likely contributes to the weak signals of Ance at the apical side (S1E Fig). Thus, erebotic cells do not acutely start to transcribe *Ance*; rather, they accumulate Ance nontranscriptionally. We hypothesized that cells may incorporate secreted Ance during erebosis. To test this hypothesis, we made a signal peptide mutant of *Ance*, and in this mutant, we did not observe erebotic cells that accumulate Ance (S7P Fig). This supports the idea that accumulation of Ance in erebotic cells is not mediated by its up-regulation but is likely mediated by incorporation of secreted one. Nontranscriptional regulation of Ance in erebotic cells is consistent with the correlation between GFP loss and Ance accumulation, where GFP loss occurs very rapidly, too rapidly to correlate with a transcriptional change.

A fundamental question is what role erebosis plays in the gut tissue homeostasis. While we were examining features of erebotic cells, we noticed that there are often small, stem cell–like cells beneath (basal to the erebotic cells) or near erebotic cells (Fig 2C). We confirmed that progenitor cells labeled by *esg>GFP* often reside close to erebotic cells (Fig 4K and 4L). Careful examination of confocal images also showed that occasionally differentiated enterocytes labeled by *Myo1D>GFP* crawl beneath erebotic cells. In one example, there was, above GFP-positive cells, strong Ance staining without DAPI, which reflects a cytoplasmic legacy without DNA after erebosis (Fig 4M). Importantly, GFP-positive cells in these observations are relatively young enterocytes because of their low expression of RFP (nlsDsRed), which takes longer maturation time than GFP [27,28]. These findings lead us to speculate that erebosis might be involved in the cell turnover mechanism in the gut. In fact, contrary to what has been believed, global inhibition of apoptosis in enterocytes with either p35 or miRNAs for *rpr*, *hid*, and *grim* did not affect ISC mitosis based on PH3 (S8A and S8B Fig). p35 did not affect EdU staining in progenitors or the number of cells lineage-traced from ISCs (S8C–S8E Fig). *dpf* mutants did not show any change of the PH3-positive cell number either (S8F Fig). Taken together, these data imply that apoptosis may not play a major role in the gut cell turnover under the physiological condition.

## Discussion

Here, we discover a previously uncharacterized phenomenon we name erebosis. Erebotic cells demonstrate accumulation of Ance, flat nuclei and loss of cytoskeleton, adhesion components, and cytoplasmic organelles. They have no classic feature of apoptosis, necrosis, or autophagy.

We consider that erebosis is a gradual process, based on the variable, gradient patterns of GFP and RFP loss and live imaging analyses. At the late stage of erebosis, TUNEL, which usually labels dying cells, becomes positive. In addition, time-lapse imaging and clonal analyses of the stem cell lineage demonstrate that erebosis readily occurs in enterocytes of the adult gut (S1 Movie and S7F–S7H Fig), while the numbers of erebotic cells remain constant in 1-, 2-, and 3-week-old flies (S1F Fig), indicating that erebotic cells continue to disappear. We also noticed that ISCs and relatively young enterocytes exist beneath or near erebotic cells. Based on these observations, we speculate that erebosis is a cell death mechanism that enables the enterocyte flux (Fig 4N).

It has been assumed that apoptosis and/or apical extrusion mediates the gut homeostasis under the physiological condition as well as stress conditions. However, at least in our culture condition, we obtained little evidence to support a main role for either of them. There is ample evidence in literature to support that apoptosis and/or apical extrusion occurs and plays important roles under stress conditions such as infection, tissue damage, and starvation [4,6,7,29]. Thus, we speculate that erebosis is mainly important in a normal physiological context. In addition, not only artificial infection, but the maintenance frequency of the fly food also affects the gut cell turnover rate [5]. In our culture system, where we change the food 3 times a week, flies are maintained in a relatively clean condition. Thus, depending on the food condition, the turnover rate, and possible mechanisms of the cell turnover could change. This could explain some differences between our study and a previous study that implicates apoptosis in normal physiological cell turnover [3].

We assume that erebosis is a molecularly coordinated process rather than accidental cell death such as necrosis. This is mainly because we reproducibly and specifically observed erebosis in the R4 region, where physiological cell turnover is the fastest. Contrary to our original expectation, we failed to find any role for Ance in erebosis itself, although it labels erebotic cells positively. In the immediate future, we will investigate molecular mechanisms by which erebosis occurs.

If we think erebosis is a type of cell death, occasionally, it seems too many cells are “dying” simultaneously under a physiological condition, implying that erebosis may not be completely inert cell death. However, in a traditional sense, due to loss of cytoskeleton, organelles, cell adhesion, and nucleic acids, it is difficult to imagine that erebotic cells are metabolically alive. Thus, it is possible that these cells may play some structural roles, such as a barrier function even if they are dying or dead. This speculation on the potential barrier function is reminiscent of the skin cell turnover where keratinizing cells lose the nucleus and organelles during cornification but maintain their barrier function in the multilayered skin tissue [30,31]. Even in a single layer tissue, cell death like erebosis might be advantageous for maintaining a state of continuous flux, since apoptosis and apical extrusion may potentially lead to breaching of the tissue integrity. Apoptosis can also potentially induce a mild level of immune reaction, albeit may not be as detrimental as necrosis. We hypothesize that erebosis is a process toward cell death, which enables the continuous flux of the gut tissue without breaching the tissue integrity or arousing immune responses.

## Methods

### Fly husbandry

Flies were maintained as previously described [32]. They were kept in vials or bottles on standard fly food containing 0.8% agar, 10% glucose, 4.5% corn flour, 3.72% dry yeast, 0.4% propionic acid, and 0.3% butyl p-hydroxybenzoate. Crosses and experiments were conducted in 25°C incubators. Collected virgins were flipped to new food at least 3 times per week. For most experiments, 5 to 9 days virgin female flies were used. Male virgins were 6 to 7 days old (S1H Fig).

### *Drosophila* stocks

The following stocks were used in this study:

*Oregon R*

*w*^*1118*^ [33]

*w-; esg-Gal4*, *UAS-GFP*, *tub-Gal80*^*ts*^ (a gift from Dr. Norbert Perrimon)

*Myo1D-Gal4*

*w-; Myo1D-Gal4*

*w-; Myo1D-Gal4, UAS-GFP/CyO*

*UAS-dcr2; act-Gal4/CyO, dfd-YFP*

*w-; tub-Gal4/TM6B*

*w-; CB-Gal4*, *UAS-mCD8-GFP* (a gift from Dr. Bruno Lemaitre)

*Npc1b-Gal4* (a gift from Dr. Leo J Pallanck)

*Ance-Gal4* (from BDSC, 76676)

*tub-Gal80*^*ts*^ (from BDSC, 7018)

*y-*, *w-; cad*::*GFP* (a gift from Dr. Takefumi Kondo)

*UAS-GFP* [34]

*UAS-DsRed* (from BDSC 6282)

*UAS-mCherry*::*mito* (from BDSC, 66533)

*UAS-nlsmCherry* (from BDSC, 38424)

*UAS-nlsRFP* (from BDSC, 8546)

*mCherry*::*Atg8a* (a gift from Dr. Eric Baehrecke)

*dpf*^*K1*^ (*Dark*^*k11502*^) (a gift from Dr. Erina Kuranaga)

*UAS-p35* (from BDSC, 5072)

*UAS-miRGH [UAS-microRNA for rpr*, *grim*, *hid]* (a gift from Dr. Iswar Hariharan)

*UAS-rpr* (from FlyORF, F002465)

GTrace [w-; UAS-FLP, UAS-nlsRFP, ubi>stop>GFP]

*esg-lexA*::*HG* (from BDSC, 66630, 66632, 66659)

*lexAop-Flp*, *ubi>>nlsGFP* (a gift from Dr. Melanie Worley and Dr. Iswar Hariharan)

*Ance*^*Δ110*^ [outcrossed to OregonR] (original stock was a gift from Dr. Kwang-Wook Choi)

*control* ^*110*^ [respective control from the last outcross of *Ance*^*Δ110*^]

*Ance*^*Δ128*^ [outcrossed to OregonR] (original stock was a gift from Dr. Kwang-Wook Choi)

*control* ^*128*^ [respective control from the last outcross of *Ance*^*Δ128*^]

*Ance-RNAi 1* (from VDRC, v41219)

*Ance-RNAi 2* [30535-3-1] (this paper)

*Ance-RNAi 3* [30535-2-1] (this paper)

*y-*, *w-; Ance-RNAi 2* [outcrossed to *y-*, *w-*]

*y-*, *w-*, *control 2* [respective control from the last outcross *Ance RNAi 2*]

*y-*, *w-; Ance-RNAi 3* [outcrossed to *y-*, *w-*]

*y-*, *w-*, *control 3* [respective control from the last outcross *Ance-RNAi 3*]

*Atg1 RNAi* (from BDSC, 26731)

*Atg2 RNAi* (from BDSC, 27706)

*Atg5 RNAi* (from BDSC, 26731)

*Atg6 RNAi* (from BDSC, 28060)

*Atg9 RNAi* (from BDSC, 28065)

*Atg12 RNAi* (from BDSC, 27552)

*Atg18a RNAi* (from BDSC, 28061)

*Drep4 RNAi* (from VDRC, v30479 and from BDSC, 67883)

*Relish RNAi* (a gift from Dr. Fumiaki Obata)

*UAS-JNK DN* (a gift from Dr. Iswar Hariharan)

*UAS-puc* (FlyORF F002209)

*hs-flp*, *tub-Gal4*, *UAS-nlsGFP;; tub-Gal80*, *FRT2A* (a gift from Dr. Yuichiro Nakajima)

*FRT2A*, *FRT82B* (a gift from Dr. Yuichiro Nakajima)

*H99*, *FRT2A* (a gift from Dr. Yuichiro Nakajima)

*DroncΔ*, *FRT2A* (a gift from Dr. Yuichiro Nakajima)

*mCherry RNAi* (from BDSC, 35785)

*attp2* (from BDSC, 36303)

*attp40* (from BDSC, 36304)

### Generation of *UAS-shRNAi* fly stocks for *Ance*

For the design and cloning of hairpins targeting *Ance* mRNA into VALIUM20, the TRiP protocol by Jian-Quan Ni and Norbert Perrimon at Harvard Medical School (https://fgr.hms.harvard.edu/files/fly/files/2ndgenprotocol.pdf) was followed, and the plasmids were sent to BestGene for transformation at attp2.

In brief, the coding CDS of Ance-RA was used for best sequence matching. Sequences with the fewest off-target effects were used to design the following oligos without 2nt shift:

shRNA-Ance-30535-2.1

ctagcagtCACCGACGATGTGCGCATCAAtagttatattcaagcataTTGATGCGCACATCGTCGGTGgcg

shRNA-Ance-30535-2.2

aattcgcCACCGACGATGTGCGCATCAAtatgcttgaatataactaTTGATGCGCACATCGTCGGTGactg

shRNA-Ance-30535-3.1

ctagcagtTTCCTGCAGTATCAACACCAAtagttatattcaagcataTTGGTGTTGATACTGCAGGAAgcg

shRNA-Ance-30535-3.2

aattcgcTTCCTGCAGTATCAACACCAAtatgcttgaatataactaTTGGTGTTGATACTGCAGGAAactg

### Generation of the Ance signal peptide mutant

CRISPR-mediated mutagenesis was performed by WellGenetics using modified methods of the published one [35]. In brief, the gRNA sequence TTTGGCGGTAACCCAAGCGC [TGG] was cloned into the U6 promoter plasmid. Cassette PBacDsRed, which contains 3xP3 DsRed flanked by PiggyBac terminal repeats, and 2 homology arms were cloned into pUC57 Kan as the donor template for repair. *Ance*/CG8827 targeting gRNAs and hs-Cas9 were supplied in DNA plasmids, together with the donor plasmid for microinjection into embryos of control strain *w*^*1118*^. F1 flies carrying selection marker of 3xP3 DsRed were further validated by genomic PCR and sequencing. CRISPR generates a 48-bp deletion in *Ance*/CG8827 deleting signal peptide (R2 to A17) of *Ance*/CG8827 and is replaced by cassette PBacDsRed. After confirming an insertion of the transgene, 3xP3-DsRed was excised by PiggyBac transposition.

### Immunohistochemistry

Midguts of adult female virgins (5 to 8 days old unless noted otherwise) were dissected in 1xPBS and fixed for 60 minutes at RT in 1xPBS with 4% paraformaldehyde (Thermo Fisher Scientific, 43368), occasionally with 10% TCA on ice (junction staining). Samples were washed 3 times for >30 minutes in PBSTx (1x PBS + 0.1% Triton X-100) and then incubated with the primary antibody in 10% NGS (Wako 143–06561, Sigma, #G9023-10ML), PBSTx overnight at 4°C. The samples were washed 3 times for >60 minutes in PBSTx before incubation with the secondary antibody in 10% NGS, PBSTx overnight at 4°C. Finally, samples were washed 5 times in PBST and mounted in Slow Fade Diamond Antifade Mountant (Invitrogen, S36963). Fluorescent images of the whole or posterior midgut (R4) were acquired using a Zeiss LSM 880 or Zeiss LSM 900 confocal microscope at 20x and 40x magnification.

The following antibodies and fluorescent dyes were used:

Polyclonal rabbit-anti-Ance (1:1,000, a gift from Dr. R. Elwyn Isaac),

mouse-anti-Armadillo (1:100, DSHB, N2 7A1),

mouse-anti-Discs large (1:100, DSHB, 4F3),

mouse-anti-LaminB1 (1:50, DSHB, ADL84.12)

mouse-anti-KDEL (1:100, MBL, M181-3)

rabbit-anti-GM130(1:100, Abcam, ab30637)

rabbit-anti-phospho-H3 (1:200, Merck, 06–570),

rabbit-anti-cleaved-Drosophila Dcp-1 (1:100, Cell Signaling, #9578),

rabbit-anti-Fibrillarin antibody (1:100, Abcam, #ab5821),

rabbit-anti-beta Tubulin antibody (1:100, Abcam, #ab179513),

Alexa Fluor phalloidin (1:100, A22287),

Alexa Fluor GFP-Booster (1:200, Chromotek, gb2AF488-50)

Alexa Fluor secondary antibodies (1:500, A11008, A11036, A32723),

DAPI (1:500, Sigma, D9542),

Hoechst 33342 (100 μg/ml, Thermo Fisher Scientific, H1399)

Propidium iodide (100 nM, Dojindo)

### Quantification of immunohistochemistry

Fiji and Zeiss ZEN were used for image analyses. The nuclear area was measured using maximum-intensity projections, and the nuclear height was measured on resliced images with automatic output spacing. For fluorescence signal quantifications, the mean or median intensity of a cell was measured and the background signal was subtracted.

### Cell counting and distance measurement

The Fiji CellCounter plugin was used for cell counting of total cell numbers and/or cell subpopulations. Therefore, images were converted to maximum-intensity projections and cells were counted with reference to the respective stacked image for inclusion of the 3D tissue organization. The distance of progenitor cells to erebotic cells was measured between cell membranes. For this experiment, expression of GFP in *esg*-expressing cells was induced in a temperature-dependent manner using the *Gal80*^*ts*^ system. Virgin female flies were collected for up to 5 days in 18°C incubators (permissive temperature) and shifted to 30°C incubators (restrictive temperature) Flies were kept at 30°C for 7 days, dissected, and imaged as described above.

### MARCM clone induction

*hs-flp*, *tub-Gal4*, *UAS-nlsGFP;; tub-Gal80 FRT2A* female flies were crossed to *H99 FRT2A*, *DroncΔ FRT2A* or *FRT2A* males to generate offspring with desired genotypes. Newly eclosed virgin female flies were kept in standard fly food for 3 to 5 days before clone induction. Then, flies were heat-shocked at 37°C for 1.5 hours in a water bath to induce clonal cells in the midgut. Intestines were dissected and stained with the anti-Ance antibody 14 days after the heat shock to examine the existence of erebotic cells in apoptosis-deficient cells.

### Propidium iodide feeding and short-term live imaging

Virgin female flies were collected for 1 to 2 days and kept on standard food as described above until becoming 6 to 8 days old. Flies were fed with 100 nM propidium iodide in 5% sucrose solution provided on a paper filter in a vial for 4 to 5 hours in a 25°C incubator. Midguts were quickly dissected in 1xPBS and incubated with 100 μg/ml Hoechst 33342 for 10 minutes. Samples were washed at least 2 times in drops of 1xPBS by transferring midguts carefully. After mounting the samples with space holders in 1xPBS, confocal imaging was performed immediately for a maximum time of 1 hour after mounting.

### Biotracker ATP-Red imaging

Biotracker ATP-Red (Sigma, SCT045), which was developed as a chemical ATP probe [36], was used to detect ATP in the gut. The live gut of *Myo1D>GFP* was embedded in 1% low melting agarose, was incubated in PBS with 10 μM BioTracker ATP-Red for 10 minutes, and was live imaged with the Zeiss LSM 900 confocal microscope.

### Time-lapse imaging

Midguts were dissected from 5- to 7-day-old female flies in PBS. The dissected midguts were embedded in 2% low melting agarose and were incubated in Schneider’s medium (ThermoFisher) supplemented with 10% FBS (ThermoFisher). The R4 region was imaged with the Zeiss LSM 900 confocal microscope (40x magnification) every 5 minutes.

### Terminal deoxynucleotidyl transferase dUTP nick end labeling (TUNEL) assay

The TUNEL assay was performed using the ApopTag Red in situ apoptosis detection kit (Millipore) according to the manufacturer’s instructions. Midguts of adult female virgins (5 to 8 days old unless noted otherwise) were dissected in 1xPBS and fixed for 60 minutes in 1xPBS with 4% paraformaldehyde at room temperature (RT). After fixation, samples were washed with PBS 0.1% Triton-X100 and incubated in equilibration buffer (Apop Tag kit; Millipore) for 10 seconds. Then, samples were incubated in reaction buffer (TdT enzyme; ratio 7:3; Apop Tag kit) at 37°C for 1 hour. The TdT reaction mix was replaced with stop buffer (diluted 1:34 in dH2O; Apop Tag kit) and incubated for 10 minutes at RT. Samples were then washed with PBS 0.1% Triton-X100 3 times and incubated with anti-digoxigenin antibody solution (diluted 31:34 in blocking solution; ApopTag kit) overnight at 4°C. Samples were then washed with PBS 0.1% Triton-X100 3 times again and mounted on the slide glass.

### EdU feeding assay

For the EdU feeding assay, a Click-iT EdU Alexa Fluor 555 Imaging Kit (Thermo Fisher Scientific C10338) was utilized. Female flies were transferred to the vials containing kimwipe soaked with 500 μM EdU and 5% sucrose and were kept for 24 hours. Midguts were dissected in 1xPBS and fixed for 60 minutes at RT in 1xPBS with 4% paraformaldehyde. After washing with PBST (0.1% Triton-X), EdU incorporation was visualized by Click reaction according to the manufacturer’s instruction. After washing with PBST for 5 minutes 3 times, guts were stained with Hoechst33342. Samples were washed with PBST for 5 minutes 3 times again and mounted on the slide glass.

### Bleomycin feeding

Virgin female flies were collected for 1 day and kept on standard food as described above until becoming 7 days old. Flies were transferred to control food (1.5% agar, 5% sucrose) or bleomycin food (1.5% agar, 5% sucrose, 300 mg/ml bleomycin (TCI, B3972)), dissected after 1 or 2 days, stained, and imaged.

### Sterile condition

To raise flies under the sterile condition, eggs were collected on grape plates for 6 hours. Embryos were bleached (3%) for 2 minutes, washed, and transferred to control food (1.5% agar, 10% yeast, 5% sucrose, propionic acid, p-hydroxybutyl benzoate) or sterile food, which was prepared by addition of penicillin and streptomycin. Virgin females and males were incubated with the food with/without the antibiotics and flipped 3 times a week until dissection at 6 to 7 days of age. Staining and imaging were performed as described above.

### GLEFA

The following GLEFA stock was generated:

*Myo1D-Gal4*, *esg-lexA-HG*, *lexAop-FLP*, *ubi>stop>nlsGFP; tub-Gal80*^*ts*^.

This GLEFA stock was crossed to *Oregon R*, *white*^*1118*^ or *UAS-p35*. The progeny were kept at 18°C until 5 to 7 days old after eclosion, then the temperature was shifted to 30°C. Flies were kept at 30°C and were dissected 5 days after the temperature shift.

### Immuno-electron microscopy

Guts were dissected and fixed by immersion in 4% paraformaldehyde and 0.1% glutaraldehyde in 0.1 M phosphate buffer (PB) (pH 7.4) for 30 minutes at RT. Guts were transferred to 4% paraformaldehyde in 0.1 M PB (pH 7.4) and further incubated for 3 hours at RT. After fixation, the guts were washed 3 times with 0.1 M PB (pH 7.4) for 10 minutes and permeabilized with 0.05% Triton X-100 in 0.1 M PB (pH 7.4) overnight with gentle agitation. After blocking with 1% BSA, 0.05% Triton X-100 in 0.1 M PB (pH 7.4) for 30 minutes at RT, the samples were incubated with chicken polyclonal anti-GFP antibody (1:2,000; #600-901-215; Rockland Immunochemicals, Limerick, PA) for overnight at RT. The samples were washed 3 times with 0.05% Triton X-100 in 0.1 M PB (pH 7.4) for 10 minutes and incubated with biotinylated goat anti-chicken IgY antibody (1:1,000; #BA-9010, Vector Laboratories, Burlingame, CA) for 2 hours at RT. After 3 washes with 0.05% Triton X-100 in 0.1 M PB (pH 7.4) for 10 minutes, the samples were incubated with streptavidin-nanogold AlexaFluor488 (1:100; #7216; Nanoprobes, Yaphank, NY) for 2 hours at RT. The samples were washed 3 times with 0.05% Triton X-100 in 0.1 M PB (pH 7.4) for 10 minutes and observed under a fluorescence stereomicroscope to confirm antibody labeling. Samples were subsequently post fixed with 1% glutaraldehyde in 0.1 M PB (pH 7.4) for 10 minutes at RT and washed 3 times with deionized water for 10 minutes at RT. Silver enhancement was performed for 4 minutes at RT using HQ Silver Enhancement Kit (#2012; Nanoprobes, Yaphank, NY), according to the manufacturer’s instructions. Samples were washed 3 times with deionized water at RT and post fixed with 1% OsO4 in 0.1 M PB (pH 7.4) for 45 minutes at 4°C. After 3 washes with deionized water for 10 minutes at RT, samples were stained en bloc with 0.5% uranyl acetate for 30 minutes at RT. After 3 washes with deionized water, samples were dehydrated by graded ethanol series (65%, 75%, 85%) for 10 minutes at each concentration, further dehydrated in 95% and 99.5% ethanol for 15 minutes at each concentration, and transferred to 100% ethanol for two 15-minute incubations. Samples were incubated in 100% acetone, transferred to a 1:1 mixture of acetone/Quetol 812 resin (Nisshin EM), and incubated overnight. After 3 transfers through Quetol 812, the R4 region was bisected with a razor to expose the region of interest, and the resin was polymerized at 60°C for >48 hours. Semithin sections (0.5 mm) were cut and stained with toluidine blue to examine the sample preparations. Ultrathin sections (50 to 70 nm) were cut and mounted on 200-mesh copper grids. The sections were stained with 0.5% uranyl acetate for 3 minutes at RT and washed with deionized water. The sections were further stained with Sato’s lead solution [37] for 3 minutes at RT, washed with deionized water, and allowed to dry. Samples were observed with JEM1010 transmission electron microscopy (JEOL) at 80 kV accelerating voltage. Images were captured with Veleta CCD camera (Olympus Soft Imaging Solutions) using iTEM software (Olympus Soft Imaging Solutions). Image processing (brightness and contrast adjustments) was performed using Fiji/ImageJ 1.53c software. Apical mitochondria area fraction was quantified by measuring the ratio between apical mitochondria and cytoplasm area. Apical cytoplasm was defined by drawing a line parallel to the apical membrane through the center of the nucleus. Distance between the apical membrane and the nucleus was measured by drawing a line orthogonal to the apical membrane that intersects the center of the nucleus and measuring its length. Anti-GFP density was quantified by counting the number of dots overlaid on the gold particles. Overlaying was performed in Adobe Illustrator CC2021 (Adobe), and quantification was performed using Fiji/ImageJ 1.53c. Cells from the same sample were compared for quantification. Data were processed using Excel (Microsoft), and plots were generated using R (R Foundation).

### Defecation (blue poo) assay

Virgin female flies were collected for 2 to 3 days and kept on standard food as described above until becoming 5 to 7 days old. In order to analyze the intestinal function, single flies were flipped to transparent cuvettes (01961–00, KARTELL S.p.A.) with blue-dyed food containing 5% sucrose, 10% yeast, and 0.5% bromophenol blue for 24 hours as previously described [38]. Defecation spots were counted on scanned images (EPSON GT-9800F).

### Statistics

Statistical tests used were indicated in the figure captions. Sample sizes were determined empirically based on the observed effects.

## Supporting information

S1 Fig Ance immunostaining in the midgut epithelium. **(A**, **B)** Control stocks *control*^*110*^ (A) and *control*^*128*^ (B) show Ance staining in an enterocyte subpopulation. (**C**, **D)** Ance protein is not detectable in 2 *Ance* mutant stocks *Ance*^*Δ110*^ (C) and *Ance*^*Δ128*^ (D). (**E)** Secreted Ance protein is present outside of cells (yellow arrowhead marks cellular F-actin) on the apical/luminal side of the midgut epithelium (white arrowhead indicates luminal Ance protein clearly surrounding F-actin labeled cells). The dotted rectangle is magnified at the left bottom. (**F)** Quantification of the number of Ance+ enterocytes in the R4 region over time. (**G)** Ance+ enterocytes are present in the R4 region of a 60-day-old fly. (**H)** This picture is the male midgut, which shows a similar expression pattern of Ance to the female one. The rest of pictures in the paper are all female guts. (**I)** Some Ance+ enterocytes show weak or absent genome staining (arrowhead) by Hoechst 33342. (**J)** Nuclear LaminB1 is absent on the nucleus of Ance+ cells depicted in the single-plane image and the orthogonal projection (arrowhead). (**K)** The orthogonal view of Phalloidin staining shows a drastic reduction of F-actin in Ance+ enterocytes (arrowhead). (**L-N)** Labeling of cell adhesion components by Dlg (septate junctions), β-catenin/Arm (adherens junctions) or cad::GFP shows reduced signals in Ance+ enterocytes in the single-plane image and the orthogonal projection (arrowhead). Statistical significance was determined by using one-way ANOVA with Dunnett’s multiple comparisons (F). S1 Data provides the source data used for all graphs and statistical analyses. Scale bars, 20 μm (A-E, H-N), 50 μm (G). Arm, Armadillo; Dlg, Discs large.
(PDF)

S2 Fig GFP fluorescence is absent in Ance+/erebotic enterocytes. **(A-C)** Ance+ cells show an inverse relationship with GFP fluorescence driven by 3 different *Gal4*-lines, *actin-Gal4* (A), *tubulin-Gal4* (B), and the mainly posterior midgut-specific *Npc1b-Gal4* (C). (**D)** In addition to GFP, Ance+ entrocytes show decreased RFP signals. (**E)** Quantification of GFP and RFP signals in normal enterocytes and erebotic cells reveals a significant reduction of both fluorescent proteins in erebotic cells. Statistical significance was determined by using a two-tailed unpaired *t* test (E). S1 Data provides the source data used for all graphs and statistical analyses. Scale bars, 20 μm.
(PDF)

S3 Fig Apoptosis inhibition does not prevent erebosis. **(A, B)** Immunostaining of Ance marks erebotic cells, which are negative for *Myo1D*-driven GFP fluorescence and can be observed in control (A) as well as with inhibition of apoptosis by ectopic p35 expression in the midgut (B). (**C)** Quantification of the percentage of erebotic cells in control and p35 expression, shown in A and B. (**D, E)** Ance+ cells are present in the midgut of *dpf*^*K1*^ (*Dark*^*k11502*^) mutant flies at the age of 2 days (G) and 20 days (H). (**F-H)** Induction of homozygous clones by MARCM for *H99* or the *Dronc* null mutation shows the presence of Ance protein in clone cells that are weakly labeled by nlsGFP (arrowheads), indicating that apoptosis-deficient mutant clones undergo erebosis. Statistical significance was determined by using a two-tailed unpaired *t* test (C). S1 Data provides the source data used for all graphs and statistical analyses. Scale bars, 20 μm.
(PDF)

S4 Fig Erebosis is different from infection-induced cellular shedding. **(A)** The infection-inducible cellular shedding reporter CB, which reflects *upd2* induction, does not drive expression of *nls-mCherry* in erebotic cells labeled by Ance immunostaining. (**B-E)** Manipulation of JNK or IMD pathway components such as expression of *JNK*^*DN*^ (C) or *puc* (D) and knockdown of *relish* (E) does not suppress the presence of erebotic cells. (**F)** Quantification of blocking JNK or IMD pathways as shown in B-E. (**G-J)** Similar to control conditions (G-H), erebotic cells can be observed in flies raised under the sterile condition in females (I) and males (J). Statistical significance was determined by using one-way ANOVA with Dunnett’s multiple comparison (F). S1 Data provides the source data used for all graphs and statistical analyses. Scale bars, 20 μm.
(PDF)

S5 Fig Autophagy is dispensable for erebosis. **(A-H)** Erebotic cells are present upon knockdown of *Atg* genes, indicating that autophagy is not required for erebosis. (**I)** Quantification of the percentage of erebotic cells upon enterocyte specific *Atg* gene knockdown indicates that blocking autophagy does not suppress erebosis. Statistical significance was determined by using one-way ANOVA with Dunnett’s multiple comparison (I). S1 Data provides the source data used for all graphs and statistical analyses. Scale bars, 20 μm.
(PDF)

S6 Fig Detailed characterization of erebosis. **(A, B)** Enterocyte-specific knockdown of the caspase-activated DNAse *Drep4* does not prevent nick formation in DNA of erebotic cells shown by the presence of TUNEL staining in GFP−/Ance+ cells. (**C, D)** Erebotic cells are present upon enterocyte-specific expression of *Drep4* RNAi. (**E)** Quantification of the percentage of erebotic cells shows that the *Drep4* knockdown does not induce reduction of erebosis. (**F)** Tubulin is reduced in erebotic cells. (**G)** In addition to nuclear LaminB1, erebotic cells decrease the nucleolus component Fibrillarin. Statistical significance was determined by using one-way ANOVA with Dunnett’s multiple comparison (E). S1 Data provides the source data used for all graphs and statistical analyses. Scale bars, 20 μm.
(PDF)

S7 Fig Ance is dispensable for erebosis. (**A-C)** Enterocyte-specific knockdown of *Ance* using 2 different RNAi lines (B, C) does reduce the abundance of Ance protein compared to control (A) but does not eliminate the presence of erebotic cells indicated by absence of GFP fluorescence. (**D)** Quantification of the Ance signal intensity in erebotic enterocytes with *Ance* RNAis. (**E)** Quantification of the percentage of erebotic enterocytes with *Ance* RNAis. (**F)** Quantification of the mitotic cell numbers in whole midguts identified by phospho-H3 immunostaining in 2 *Ance* mutants and their respective control lines that were either kept on control food or food supplemented with bleomycin. (**G, H)** Overexpression of *Ance* specifically in enterocytes does not affect the presence of erebotic cells. (**I)** Quantification of the percentage of erebotic cells upon overexpression of Ance shows no change in the frequency of erebosis. (**J)** *hh-Gal4*–driven *Ance* expression increases the amount of Ance protein in the posterior wing disc, indicating that the *UAS-Ance* construct is functional. (**K)** Representative images of fecal deposits on a glass cuvette after defecation assay performance using 2 different RNAi lines for enterocyte-specific *Ance* knockdown and their respective control flies. (**L)** Quantification of fecal deposits within 24 hours. (**M)** Quantification of the percentage of concentrated fecal deposits within 24 hours. *Ance* knockdown reduces the amounts of concentrated fecal deposits. (**N, O)** *Ance-Gal4* drives GFP expression in both erebotic cells and cells surrounding them in the R4. Although cells in late erebosis with high Ance have very little GFP signals (yellow arrowhead), cells in intermediate erebosis with a moderate amount of Ance have clear GFP signals (white arrowhead). (**P)** The Ance signal peptide deletion mutant, which lacks the signal peptide (R2 to A17), does not demonstrate specific patterns of Ance immunostaining, suggesting that secretion of Ance might be important for it to accumulate in erebotic cells. Statistical significance was determined by using a two-tailed paired *t* test (F, I, L, M) or one-way ANOVA with Dunnett’s multiple comparison (D, E). S1 Data provides the source data used for all graphs and statistical analyses. Scale bars, 20 μm (A-C, G, O, P), 50 μm (J, N).
(PDF)

S8 Fig Apoptosis inhibition does not prevent ISC proliferation. **(A**, **B)** Quantification of the mitotic cell numbers in whole midguts identified by phospho-H3 immunostaining of 3 different control conditions (2 TRiP control lines and *mCherry* RNAi) and 2 constructs that inhibit apoptosis expressed in enterocytes (ectopic expression of *p35* or microRNAs against *rpr*, *grim*, and *hid*). Flies were kept either on control food (A) or on food supplemented with bleomycin (B) for 1 day. (**C)** Quantification of EdU-incorporated progenitor in posterior midguts. Note that we excluded Edu-positive endoreplicating enterocytes from counting. (**D)** A schematic of GLEFA system, which enables both gene manipulation in enterocytes and lineage tracing from progenitor cells. (**E)** Lineage tracing by GLEFA demonstrates that *p35* expression in enterocytes does not affect proliferation of progenitor cells. (**F)** Quantification of the mitotic cell numbers in whole midguts identified by phospho-H3 immunostaining in *dpf*^*K1*^ (*Dark*^*k11502*^) mutant and heterozygous control. Statistical significance was determined by using a two-tailed unpaired *t* test (C, F) or one-way ANOVA with Dunnett’s multiple comparison (E). S1 Data provides the source data used for all graphs and statistical analyses. GLEFA, Gal4-LexA Enterocyte Flux Analysis; ISC, intestinal stem cell.
(PDF)

S1 Movie Time-lapse imaging of erebosis (Myo1D>GFP, nlsRFP). (MP4)

S1 Data Source data. The numerical values used for all main and supporting figures are provided.
(XLSX)

## Abbreviations

Ance: Angiotensin-converting enzyme
ISC: intestinal stem cell
immuno-EM: immuno-electron microscopy
PB: phosphate buffer
RT: room temperature
