# Supplementary figures and images for: Erebosis, a new cell death mechanism during homeostatic turnover of gut enterocytes
Source: PLoS Biol. 2022 Apr 25;20(4):e3001586. doi: 10.1371/journal.pbio.3001586 (PMC9037934; doi:10.1371/journal.pbio.3001586)

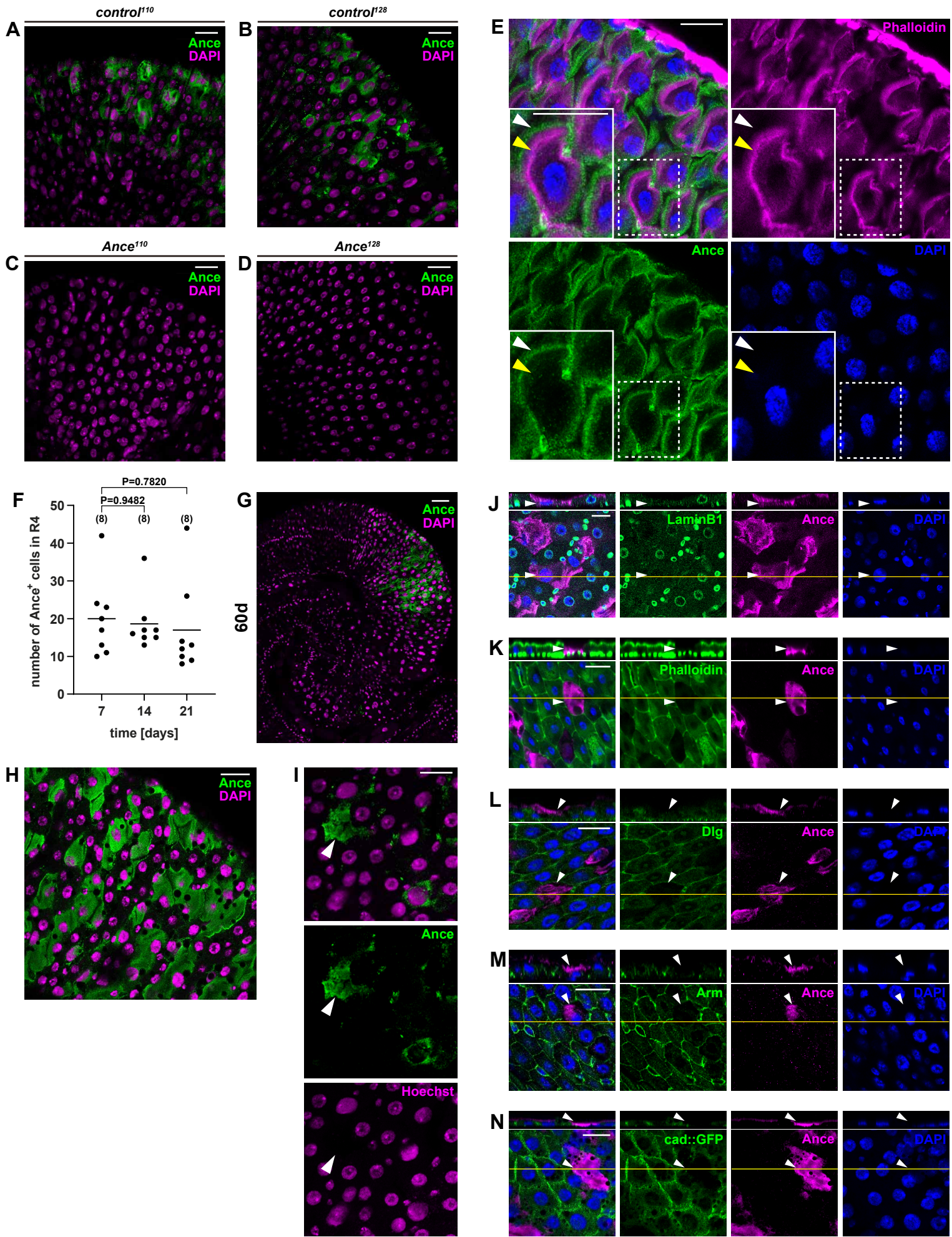

Supplement: S1 Fig — (A, B) Control stocks control110 (A) and control128 (B) show Ance staining in an enterocyte subpopulation. (C, D) Ance protein is not detectable in 2 Ance mutant stocks AnceΔ110 (C) and AnceΔ128 (D). (E) Secreted Ance protein is present outside of cells (yellow arrowhead marks cellular F-actin) on the apical/luminal side of the midgut epithelium (white arrowhead indicates luminal Ance protein clearly surrounding F-actin labeled cells). The dotted rectangle is magnified at the left bottom. (F) Quantification of the number of Ance+ enterocytes in the R4 region over time. (G) Ance+ enterocytes are present in the R4 region of a 60-day-old fly. (H) This picture is the male midgut, which shows a similar expression pattern of Ance to the female one. The rest of pictures in the paper are all female guts. (I) Some Ance+ enterocytes show weak or absent genome staining (arrowhead) by Hoechst 33342. (J) Nuclear LaminB1 is absent on the nucleus of Ance+ cells depicted in the single-plane image and the orthogonal projection (arrowhead). (K) The orthogonal view of Phalloidin staining shows a drastic reduction of F-actin in Ance+ enterocytes (arrowhead). (L-N) Labeling of cell adhesion components by Dlg (septate junctions), β-catenin/Arm (adherens junctions) or cad::GFP shows reduced signals in Ance+ enterocytes in the single-plane image and the orthogonal projection (arrowhead). Statistical significance was determined by using one-way ANOVA with Dunnett’s multiple comparisons (F). S1 Data provides the source data used for all graphs and statistical analyses. Scale bars, 20 μm (A-E, H-N), 50 μm (G). Arm, Armadillo; Dlg, Discs large. (PDF) [file pbio.3001586.s001.pdf]

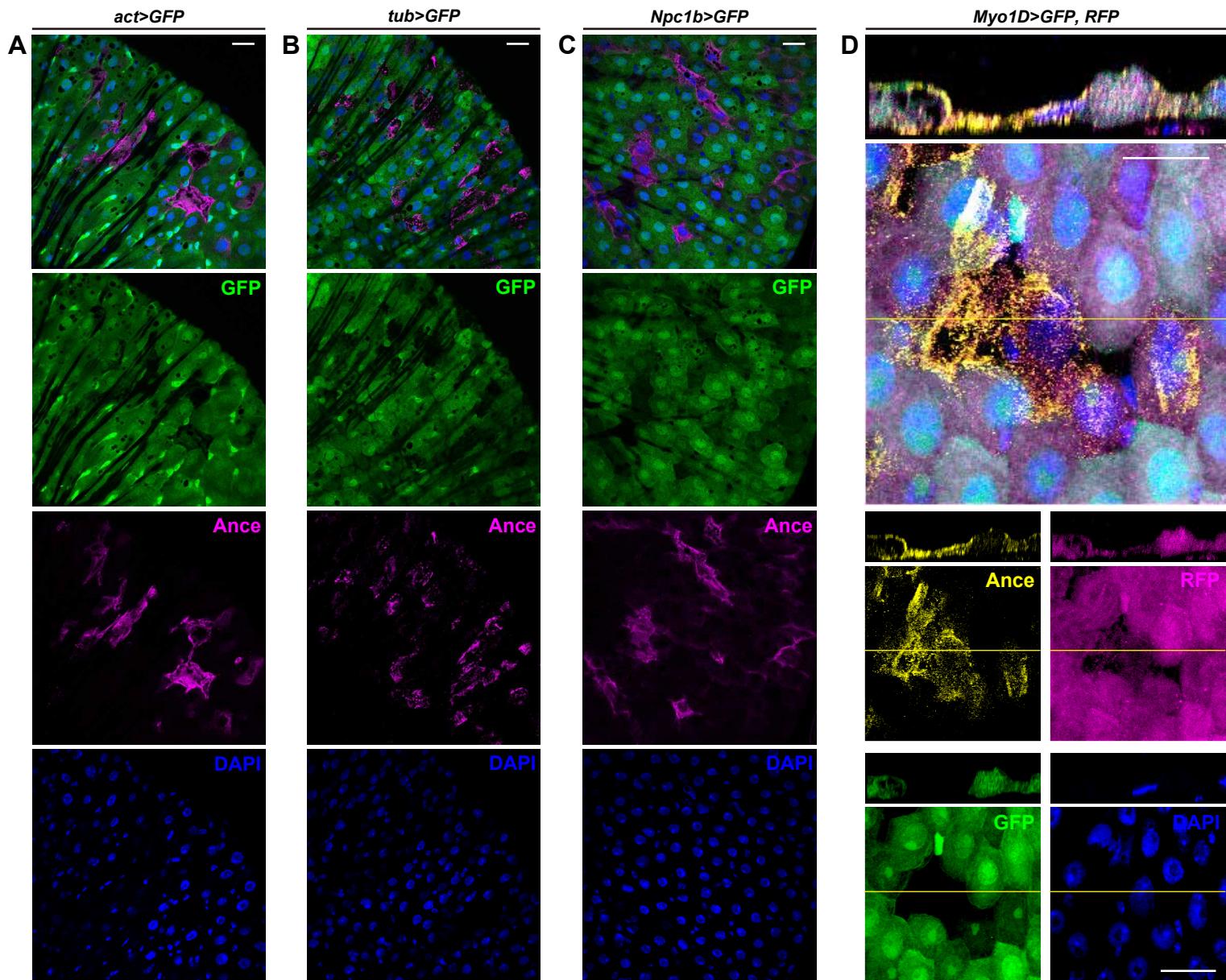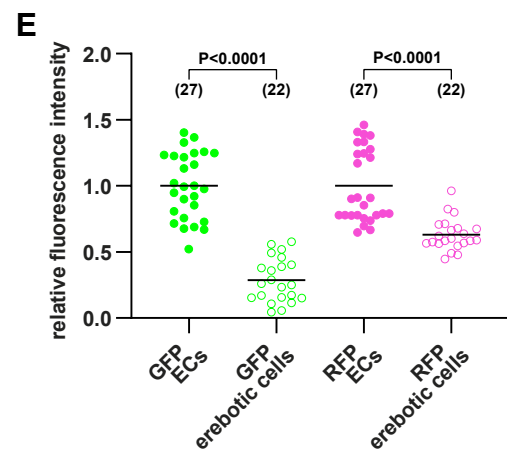

Supplement: S2 Fig — (A-C) Ance+ cells show an inverse relationship with GFP fluorescence driven by 3 different Gal4-lines, actin-Gal4 (A), tubulin-Gal4 (B), and the mainly posterior midgut-specific Npc1b-Gal4 (C). (D) In addition to GFP, Ance+ entrocytes show decreased RFP signals. (E) Quantification of GFP and RFP signals in normal enterocytes and erebotic cells reveals a significant reduction of both fluorescent proteins in erebotic cells. Statistical significance was determined by using a two-tailed unpaired t test (E). S1 Data provides the source data used for all graphs and statistical analyses. Scale bars, 20 μm. (PDF) [file pbio.3001586.s002.pdf]

*Myo1D>GFP*

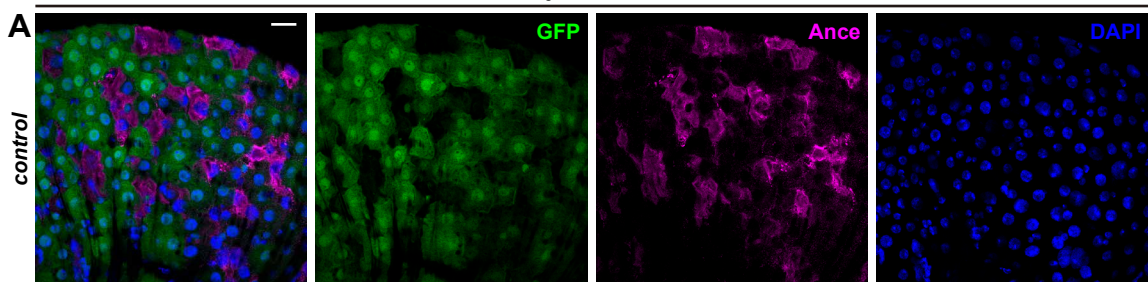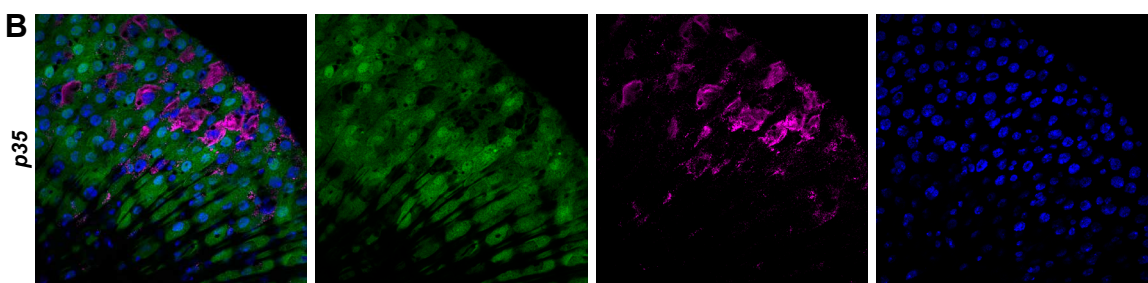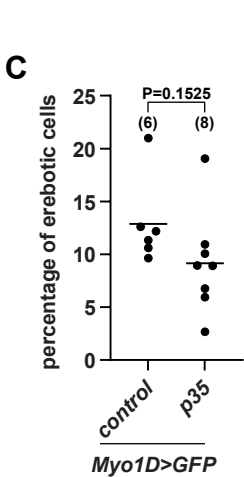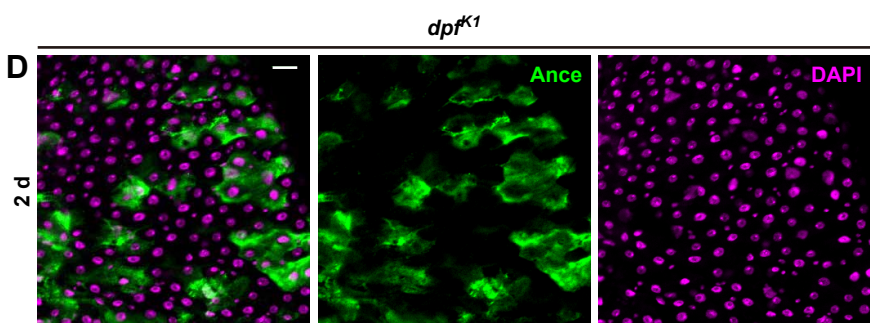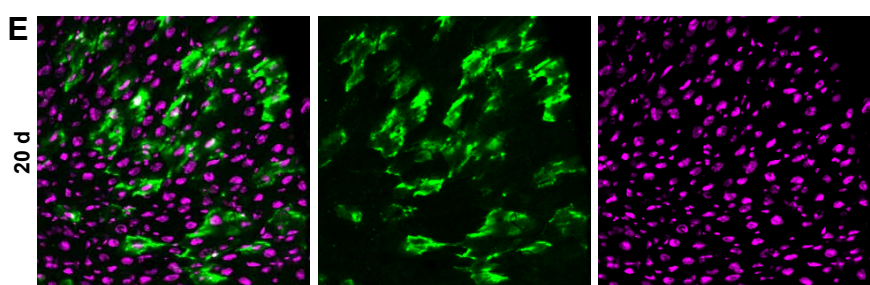

*hs-flip, tub>nlsGFP;; tub-Gal80, FRT2A*

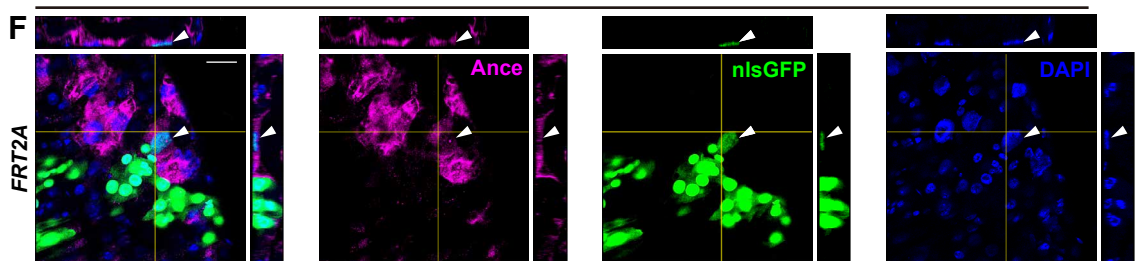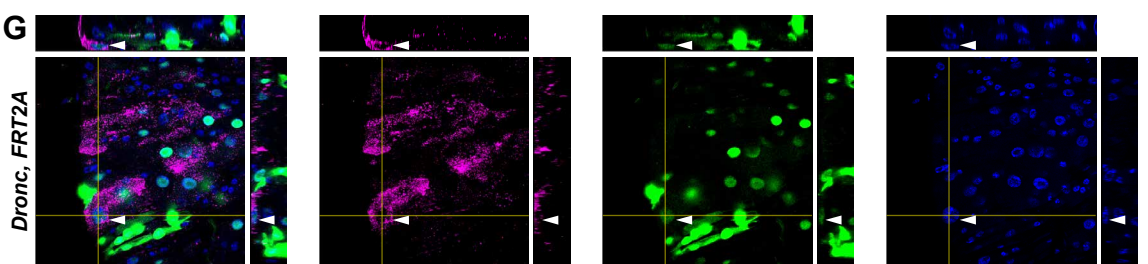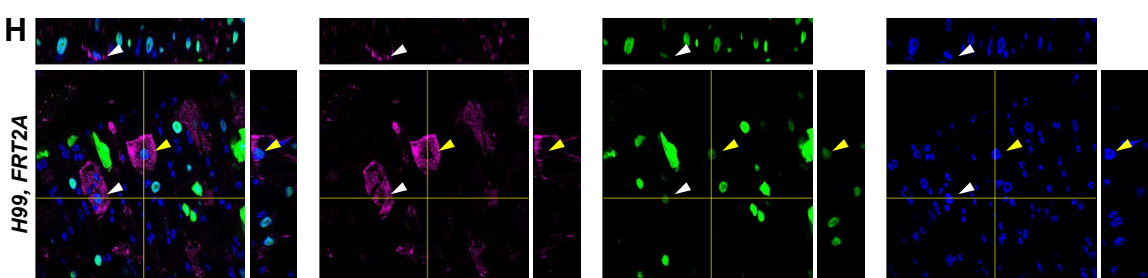

Supplement: S3 Fig — (A, B) Immunostaining of Ance marks erebotic cells, which are negative for Myo1D-driven GFP fluorescence and can be observed in control (A) as well as with inhibition of apoptosis by ectopic p35 expression in the midgut (B). (C) Quantification of the percentage of erebotic cells in control and p35 expression, shown in A and B. (D, E) Ance+ cells are present in the midgut of dpfK1 (Darkk11502) mutant flies at the age of 2 days (G) and 20 days (H). (F-H) Induction of homozygous clones by MARCM for H99 or the Dronc null mutation shows the presence of Ance protein in clone cells that are weakly labeled by nlsGFP (arrowheads), indicating that apoptosis-deficient mutant clones undergo erebosis. Statistical significance was determined by using a two-tailed unpaired t test (C). S1 Data provides the source data used for all graphs and statistical analyses. Scale bars, 20 μm. (PDF) [file pbio.3001586.s003.pdf]

*CB>nls-mCherry*

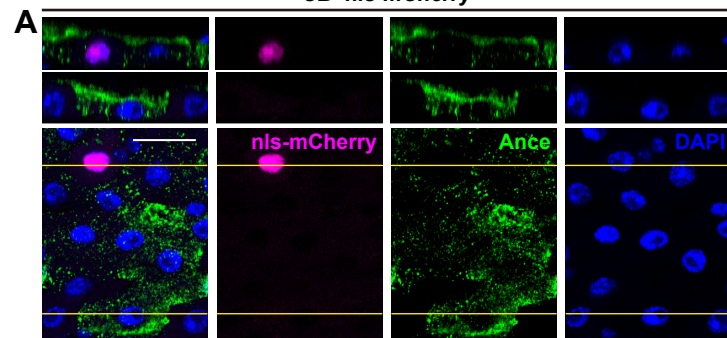

*Myo1D>GFP, nlsRFP, tub-Gal80<sup>ts</sup>*

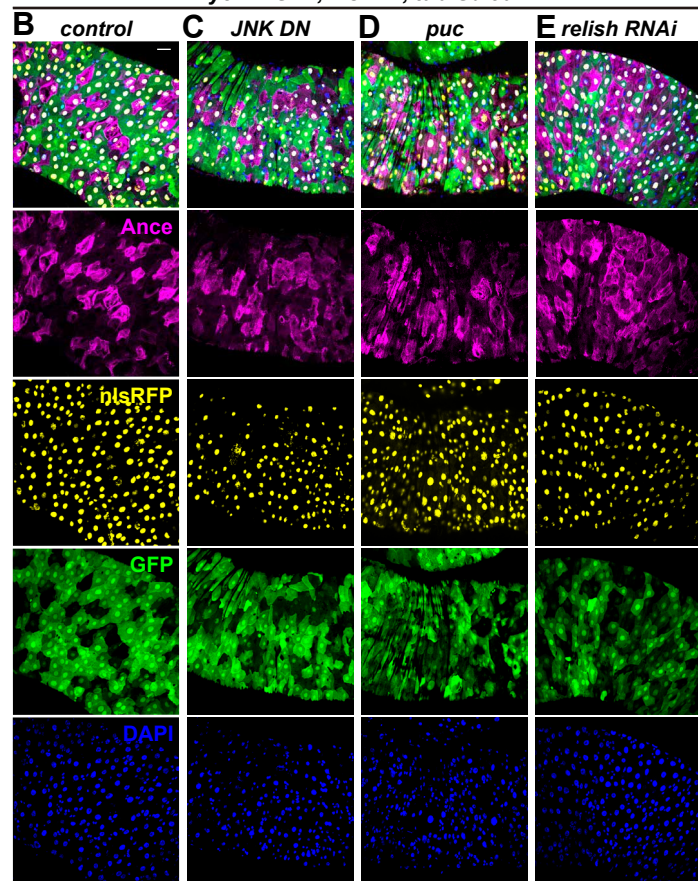

*Myo1D>GFP*

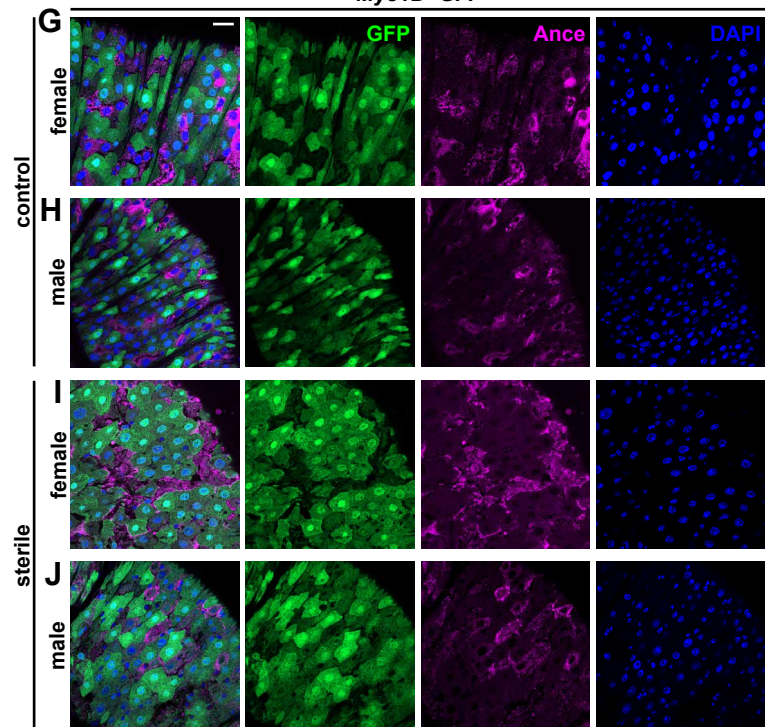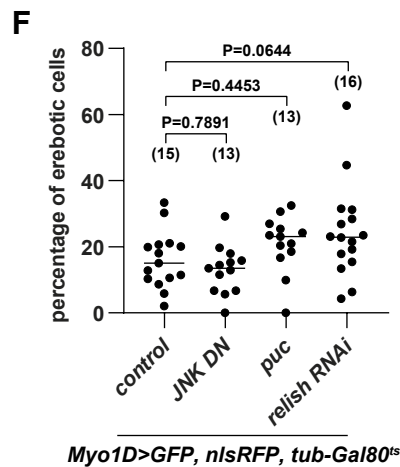

Supplement: S4 Fig — (A) The infection-inducible cellular shedding reporter CB, which reflects upd2 induction, does not drive expression of nls-mCherry in erebotic cells labeled by Ance immunostaining. (B-E) Manipulation of JNK or IMD pathway components such as expression of JNKDN (C) or puc (D) and knockdown of relish (E) does not suppress the presence of erebotic cells. (F) Quantification of blocking JNK or IMD pathways as shown in B-E. (G-J) Similar to control conditions (G-H), erebotic cells can be observed in flies raised under the sterile condition in females (I) and males (J). Statistical significance was determined by using one-way ANOVA with Dunnett’s multiple comparison (F). S1 Data provides the source data used for all graphs and statistical analyses. Scale bars, 20 μm. (PDF) [file pbio.3001586.s004.pdf]

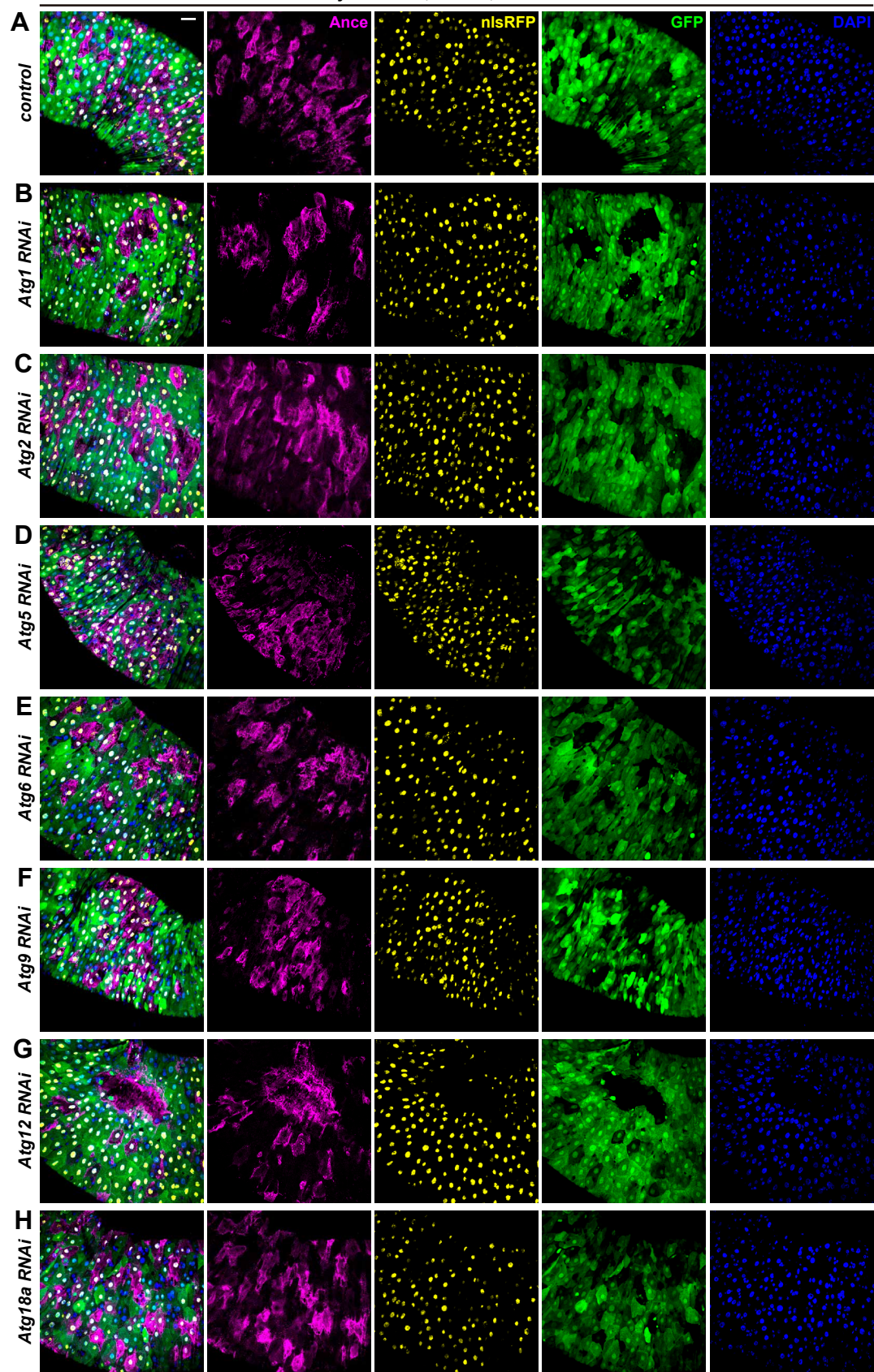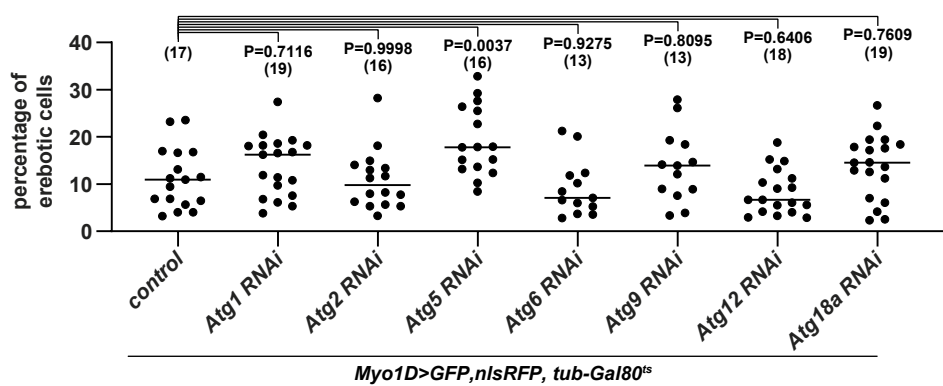

Supplement: S5 Fig — (A-H) Erebotic cells are present upon knockdown of Atg genes, indicating that autophagy is not required for erebosis. (I) Quantification of the percentage of erebotic cells upon enterocyte specific Atg gene knockdown indicates that blocking autophagy does not suppress erebosis. Statistical significance was determined by using one-way ANOVA with Dunnett’s multiple comparison (I). S1 Data provides the source data used for all graphs and statistical analyses. Scale bars, 20 μm. (PDF) [file pbio.3001586.s005.pdf]

*Myo1D>GFP, tub-Gal80<sup>ts</sup>*

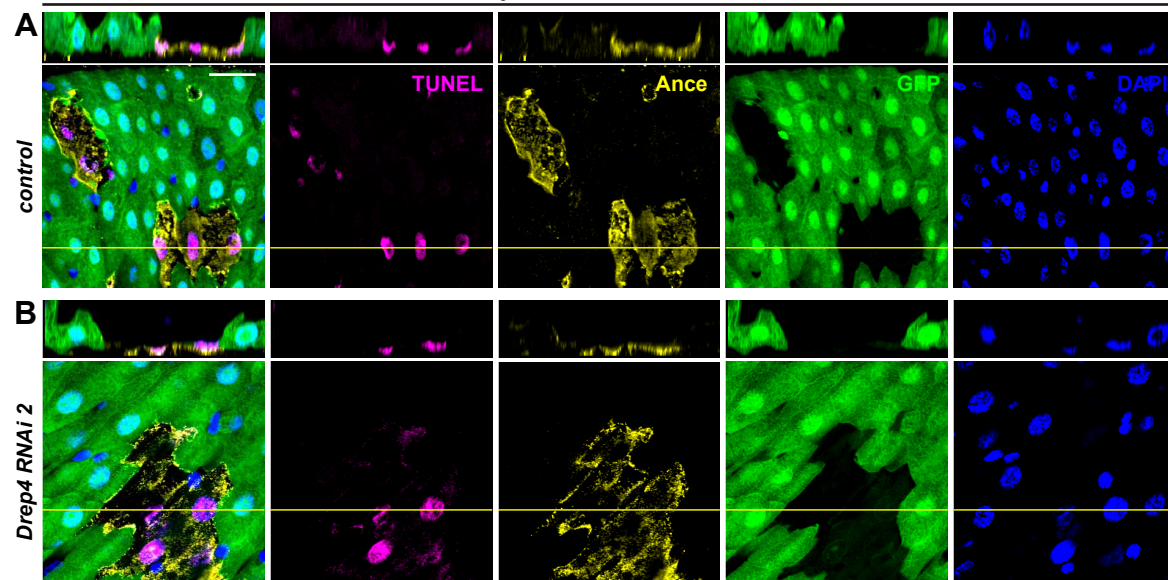

*Myo1D>GFP, nlsRFP, tub-Gal80<sup>ts</sup>*

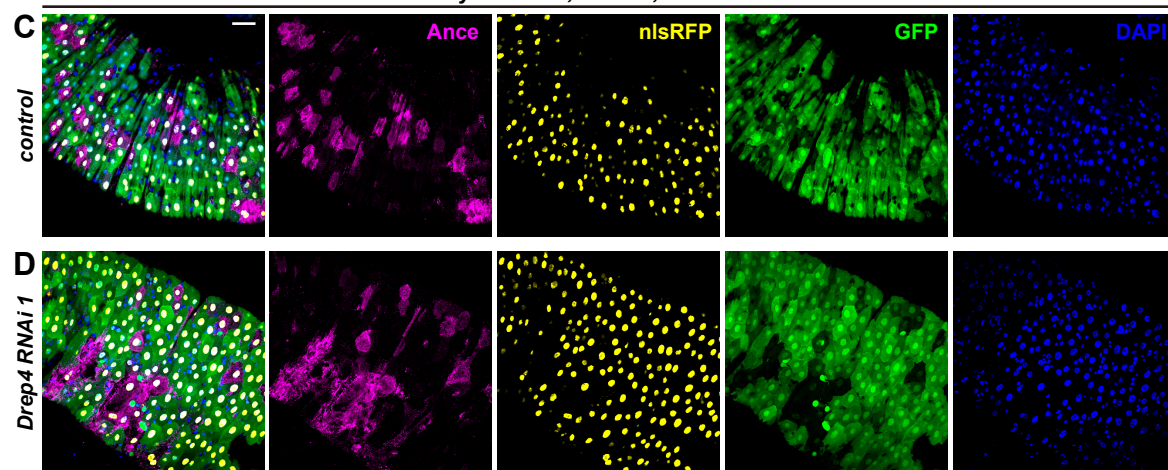

*Myo1D>GFP, nlsRFP*

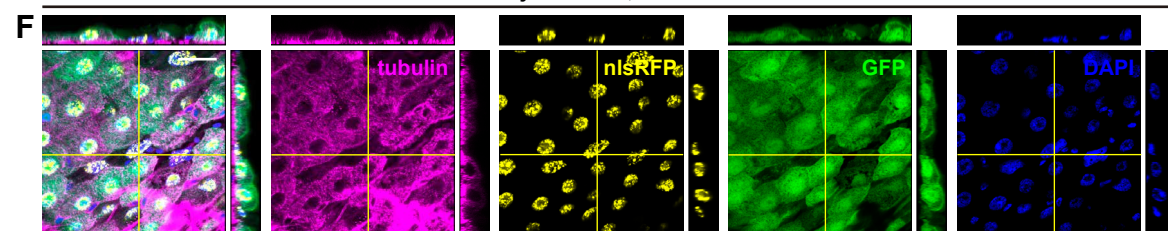

*Myo1D>GFP*

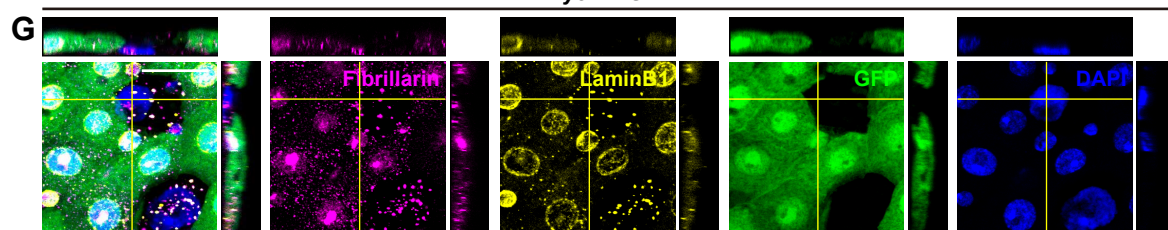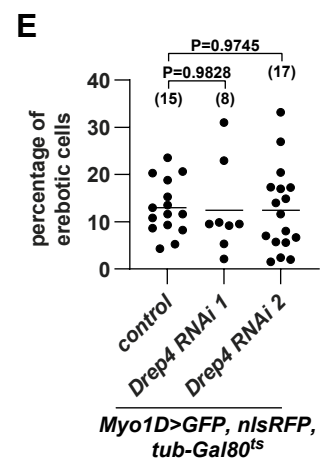

Supplement: S6 Fig — (A, B) Enterocyte-specific knockdown of the caspase-activated DNAse Drep4 does not prevent nick formation in DNA of erebotic cells shown by the presence of TUNEL staining in GFP−/Ance+ cells. (C, D) Erebotic cells are present upon enterocyte-specific expression of Drep4 RNAi. (E) Quantification of the percentage of erebotic cells shows that the Drep4 knockdown does not induce reduction of erebosis. (F) Tubulin is reduced in erebotic cells. (G) In addition to nuclear LaminB1, erebotic cells decrease the nucleolus component Fibrillarin. Statistical significance was determined by using one-way ANOVA with Dunnett’s multiple comparison (E). S1 Data provides the source data used for all graphs and statistical analyses. Scale bars, 20 μm. (PDF) [file pbio.3001586.s006.pdf]

# Myo1D>GFP

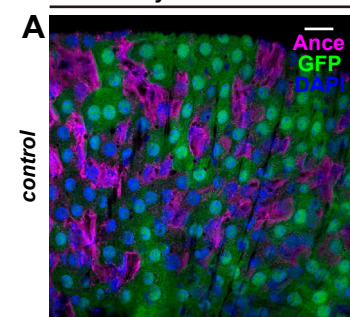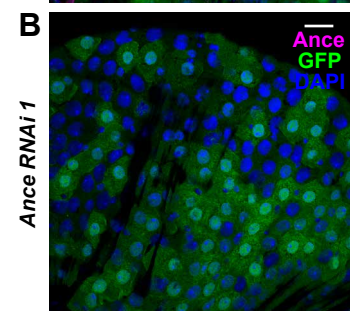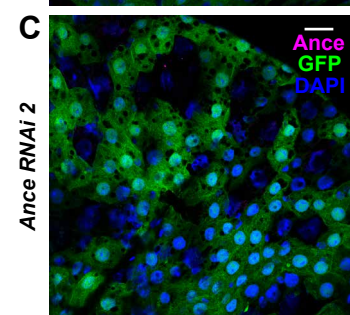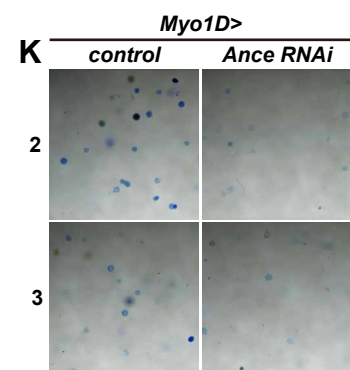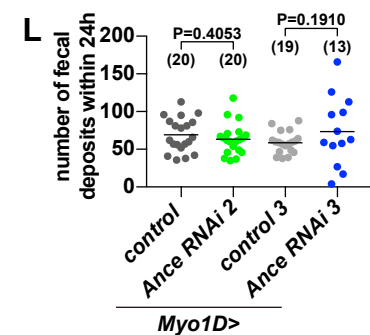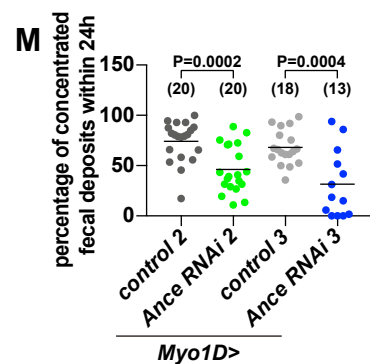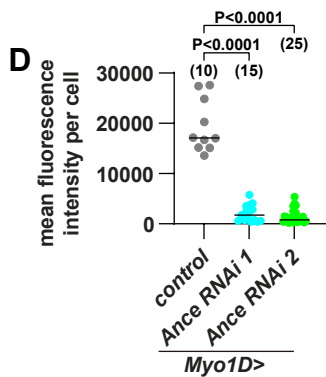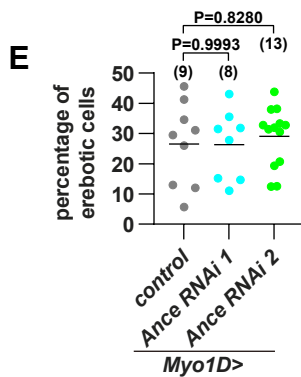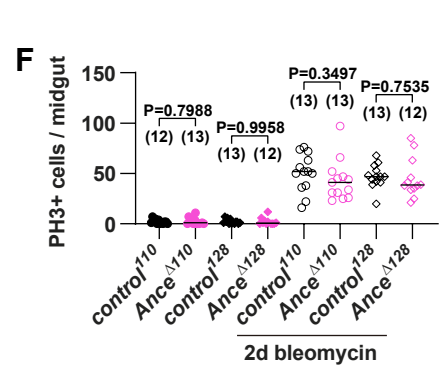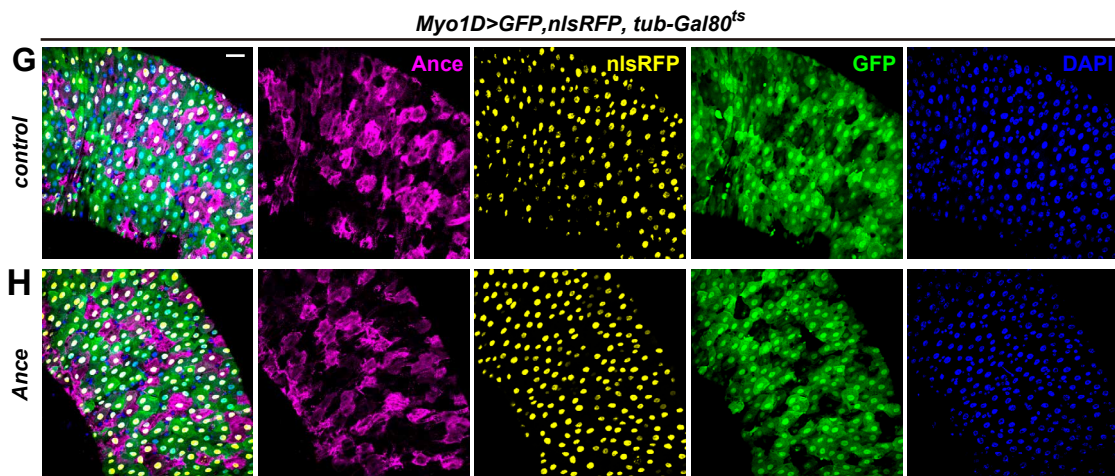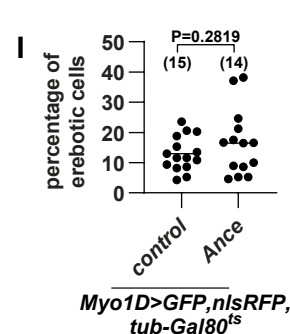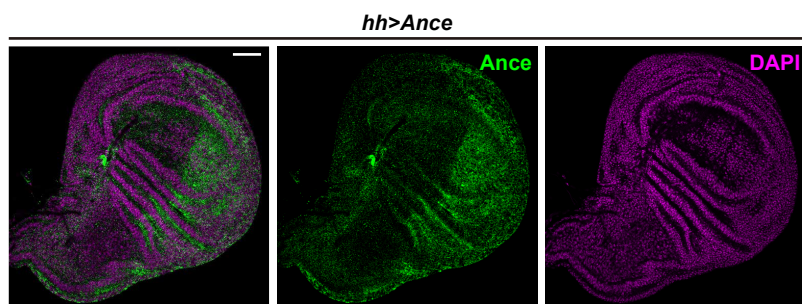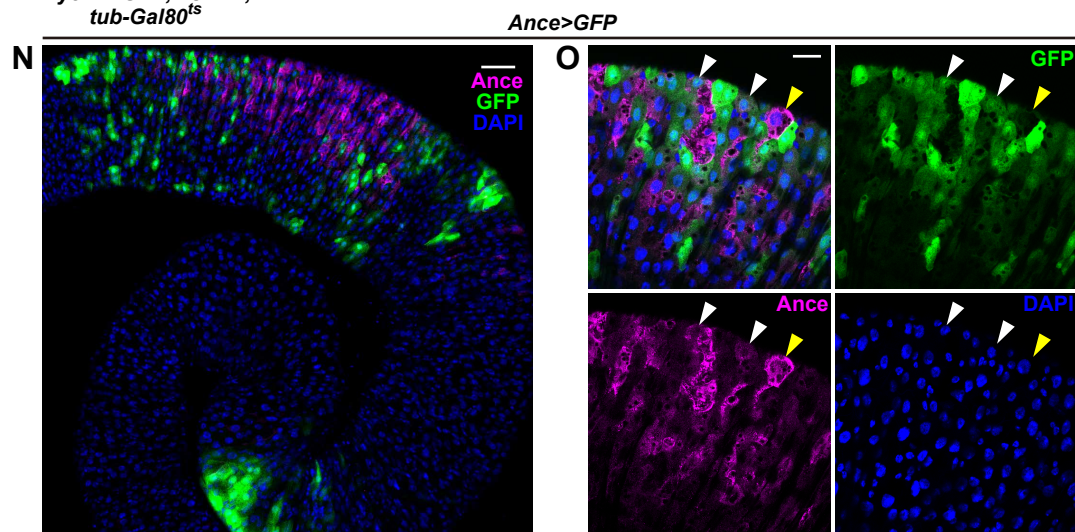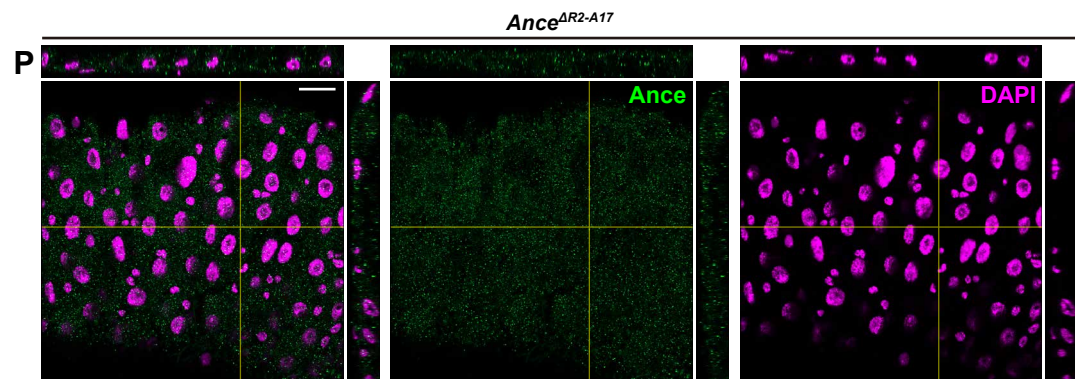

Supplement: S7 Fig — (A-C) Enterocyte-specific knockdown of Ance using 2 different RNAi lines (B, C) does reduce the abundance of Ance protein compared to control (A) but does not eliminate the presence of erebotic cells indicated by absence of GFP fluorescence. (D) Quantification of the Ance signal intensity in erebotic enterocytes with Ance RNAis. (E) Quantification of the percentage of erebotic enterocytes with Ance RNAis. (F) Quantification of the mitotic cell numbers in whole midguts identified by phospho-H3 immunostaining in 2 Ance mutants and their respective control lines that were either kept on control food or food supplemented with bleomycin. (G, H) Overexpression of Ance specifically in enterocytes does not affect the presence of erebotic cells. (I) Quantification of the percentage of erebotic cells upon overexpression of Ance shows no change in the frequency of erebosis. (J) hh-Gal4–driven Ance expression increases the amount of Ance protein in the posterior wing disc, indicating that the UAS-Ance construct is functional. (K) Representative images of fecal deposits on a glass cuvette after defecation assay performance using 2 different RNAi lines for enterocyte-specific Ance knockdown and their respective control flies. (L) Quantification of fecal deposits within 24 hours. (M) Quantification of the percentage of concentrated fecal deposits within 24 hours. Ance knockdown reduces the amounts of concentrated fecal deposits. (N, O) Ance-Gal4 drives GFP expression in both erebotic cells and cells surrounding them in the R4. Although cells in late erebosis with high Ance have very little GFP signals (yellow arrowhead), cells in intermediate erebosis with a moderate amount of Ance have clear GFP signals (white arrowhead). (P) The Ance signal peptide deletion mutant, which lacks the signal peptide (R2 to A17), does not demonstrate specific patterns of Ance immunostaining, suggesting that secretion of Ance might be important for it to accumulate in erebotic cells. Statistical signi [file pbio.3001586.s007.pdf]

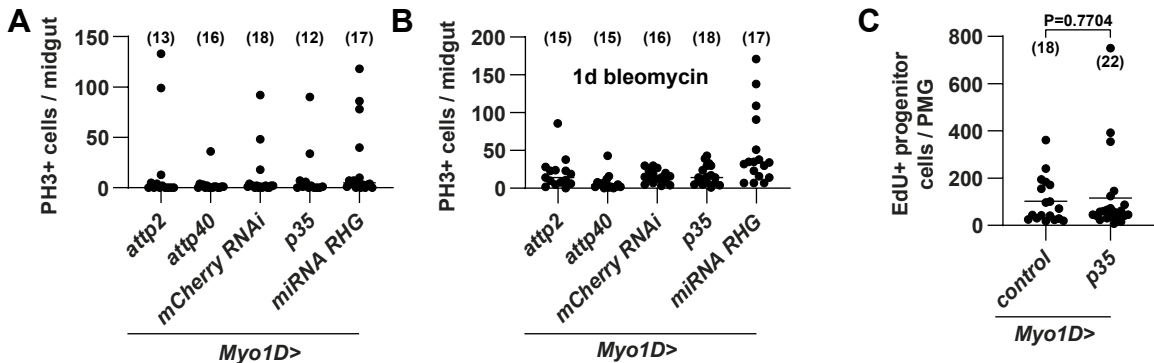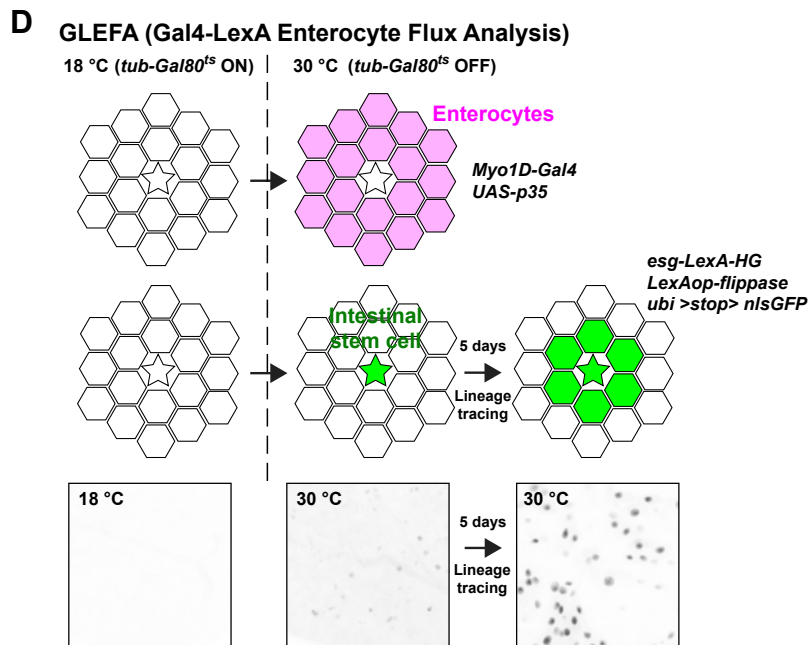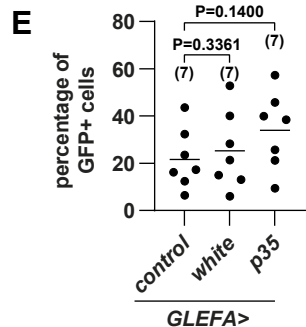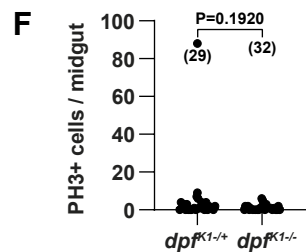

Supplement: S8 Fig — (A, B) Quantification of the mitotic cell numbers in whole midguts identified by phospho-H3 immunostaining of 3 different control conditions (2 TRiP control lines and mCherry RNAi) and 2 constructs that inhibit apoptosis expressed in enterocytes (ectopic expression of p35 or microRNAs against rpr, grim, and hid). Flies were kept either on control food (A) or on food supplemented with bleomycin (B) for 1 day. (C) Quantification of EdU-incorporated progenitor in posterior midguts. Note that we excluded Edu-positive endoreplicating enterocytes from counting. (D) A schematic of GLEFA system, which enables both gene manipulation in enterocytes and lineage tracing from progenitor cells. (E) Lineage tracing by GLEFA demonstrates that p35 expression in enterocytes does not affect proliferation of progenitor cells. (F) Quantification of the mitotic cell numbers in whole midguts identified by phospho-H3 immunostaining in dpfK1 (Darkk11502) mutant and heterozygous control. Statistical significance was determined by using a two-tailed unpaired t test (C, F) or one-way ANOVA with Dunnett’s multiple comparison (E). S1 Data provides the source data used for all graphs and statistical analyses. GLEFA, Gal4-LexA Enterocyte Flux Analysis; ISC, intestinal stem cell. (PDF) [file pbio.3001586.s008.pdf]
